# Novel benzylidene benzofuranone analogues as potential anticancer agents: design, synthesis and in vitro evaluation based on CDK2 inhibition assays

**DOI:** 10.1007/s13205-022-03312-1

**Published:** 2022-09-02

**Authors:** Aravinda Pai, Jayashree B.S.

**Affiliations:** Department of Pharmaceutical Chemistry, Manipal College of Pharmaceutical Sciences, Manipal Academy of Higher Education, Manipal, Karnataka 576104 India

**Keywords:** CDK2, Cyclin A, Flavopiridol, Benzofuranones, FRET, Anticancer

## Abstract

The classical anticancer agents do not have their efficacy on inhibiting the G2 phase of the cell cycle. There are a very few reports available on drugs that work at G2 phase. Flavopiridol is one such drug candidate. In the current study, we sought to make analogues of flavopiridol. Still, the conditions used during their synthesis were unfavourable for the formation of flavopiridol and led to the generation of benzofuranones. In the present work, a new series of benzylidene benzofuranones were designed, synthesized and evaluated for their antioxidant, anti-colorectal cancer activity. Molecular docking, MMGBSA and molecular dynamics studies were conducted to assess their binding affinity at the active site of CDK2. Based on the cytotoxicity exhibited by test compounds, the compound NISOA4 (from isopropyl series) was further selected for mechanistic anticancer studies on HCT 116 cell lines. The compound selected was evaluated by comet assay, DNA fragmentation assay, cell cycle analysis, apoptosis detection by annexin FITC, semi-quantitative RTPCR based gene expression studies and FRET assay on the target CDK2/Cyclin A. Compound NISOA4 exhibited marked olive moments in comet assay studies. The apoptotic DNA fragmentation for compound NISOA4 demonstrated a marked change in the DNA fragmentation. The compound exhibited cell cycle arrest at G2/M phase at both the test concentrations. Apoptosis induction was observed at both the test concentrations and the compound was found to be a potent proapoptotic agent. It exhibited marked inhibition for the CDK2 gene expression and did not show any effect on CyclinA gene expression. However, the compound NISOA4 along with other analogues showed appreciable inhibition for the CDK2/Cyclin A target enzyme.

**Supplementary Information:**

The online version contains supplementary material available at 10.1007/s13205-022-03312-1.

## Introduction

Cancer is a bewildering disease. There is a loss in the normal cell regulatory mechanisms that controls cell survival, differentiation and proliferation (Singh et al. 2021a, b).The primary drawback of current chemotherapeutics as anti-neoplastic agents is that monotherapy, as well as multi-drug combinations, causes drug resistance. Cross resistance is another threat for the existing drugs that has restricted the use in chemotherapy. Instead, these restrictions have supported researchers to have better insight to understand the molecular processes that underlie the onset of different stages of cancer (Jayashree et al 2015). In the light of this, much efforts are being made so as to identify and validate newer targets in cancer biology (Singh et al. 2022). Some of the anticancer targets that are presently identified in the clinical set up includes tyrosine kinase (Bhardwaj et al. 2020), Farnesyl transferases, histone deacetylases, aromatase and cyclin-dependent kinases (CDKs). CDKs (Meijer et al.^1997^) and their regulatory subunits cyclins are the key molecules that control and coordinate DNA-synthesis, chromosome separation, neuronal differentiation, apoptosis and cell division. Together, they drive the cell from one phase of the cell cycle to the next. The revelations concerning the control of the cell cycle won the noble prize for Medicine and physiology in 2001. Later, Leland Hartwell, Timothy Hunt, and Paul Nurse together conceptualized cell cycle regulation (Nurse and Bissett 1981) at the sub-atomic level concerning how the cell could be driven out starting from one stage onto the next amidst the cell division (Tyers 2004).The cell cycle specifically coordinates with all cellular processes, which includes metabolism, transcriptional and translational regulation, DNA synthesis and replication, cytoskeletal dynamics and chromosome segregation (Engh and Bossemeyer 2001).

The cell cycle contains a total of four different stages. In response to mitogenic signals, cell development progresses from the dormant phase, G0, to G1, where they normally commit to continuing through the cell cycle. Additionally, the length of the cell cycle (Lee and Nurse 1987) is solely dependent on the type of cells. Its length in the majority of mammalian cells ranges from 10 to 30 h.

The many stages of the cell cycle are carefully regulated and timed so that one stage is finished before the next one starts. Chromosomal abnormalities may result from errors that manifest in coordination. Further changes to the chromosomal structure could occur as a result of chromosomal abnormalities or certain rearrangements. Morgan (1997). This eventually led to further structural alterations in the chromosomes resulting in the onset of cancer. This type of alteration is frequently observed in malignant cells. It is known that genes associated with CDK molecules and cyclins function (Serizawa et al. 1995) are oncogenes/cancer-causing genes. CDK molecules (Cohen P 1999)  as well as cyclins are known to be associated with the products of tumor-suppressor genes during the cell cycle and their levels gets overexpressed in tumor cells.

**Fig. 1:**
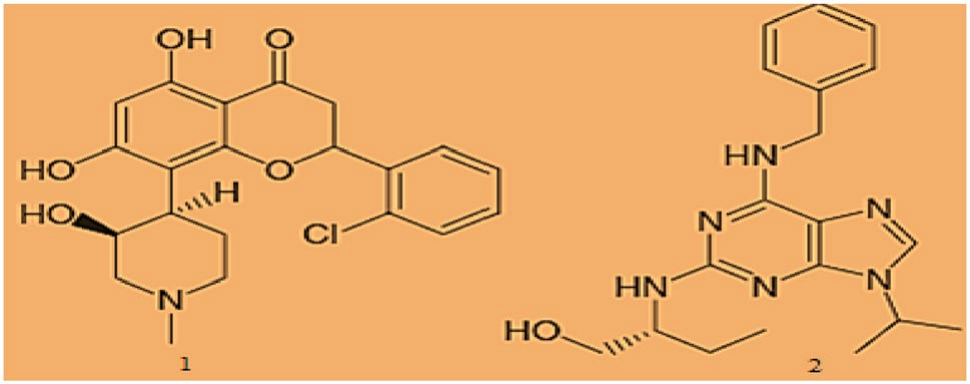
Structure of flavopiridol and roscovitine

The three CDK/Cyclin complexes required for cell division are CDK2/Cyclin E, which moves the cell across the G1/S-phase border, CDK2/Cyclin A, which encourages DNA replication during the S phase, and CDK1/Cyclin B, which controls the cell’s entry into mitosis (Ahn et al. 2007). It is interesting to notice that, the phosphate donor always forms a complex with a divalent metal ion like Mg2+ or Mn2+ . Their role is to selectively transfer the phosphoryl group into the macromolecular substrate binding site by attaching to and orienting it (Semenov et al. 2002).

In the past few decades, many other CDK2 inhibitors (Singh et al. 2021a, b) with various scaffolds (Kaur et al. 1992) have been created and developed; some of them have even gone through clinical trials because of their potential to treat cancer. As of right now, not a single drug candidate in this category has been licenced for commercial usage as an anti-cancer therapy. Given CDK2's druggable nature and frequently dysregulated expression in a variety of cancers, additional research is likely to be crucial for the development of CDK2 inhibitors as anticancer drugs. Figure 1 shows the structure of first-generation CDK2 inhibitors (structure 1 represents flavopiridol and structure 2 represents Roscovitine).

**Fig. 2:**
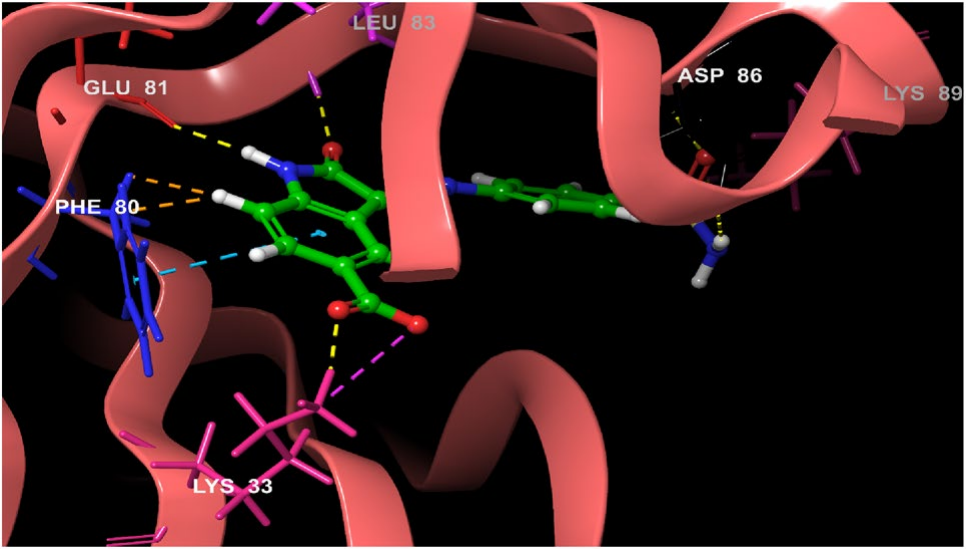
Crystal ligand interaction at the active site of CDK2/cyclin A

According to the literature, various heterocyclic compounds related to flavopiridol (Kim et al. 2000), such as those with methoxy, hydroxyl groups, and basic nitrogen moieties, have demonstrated strong anti-cancer activity in a number of studies because of their anti-mitotic, cell cycle arrest, and antioxidant effects. In the light of this, in one of our previous studies, the synthesis and anti- cancer activity of substituted N-methyl tetrahydropyridinyl chalcones, namely 1-methyl-4- (2,4,6-trimethoxyphenyl)-1,2,3,6-tetrahydropyridinyl-2ˊ-hydroxy chalcones possessing different bio-isosteric heterocyclic substituents on the ring B were reported. Literature search also revealed screening of natural product libraries against various isoforms of CDK (Singh et al. 2020). In continuation of this work, it was thought worthwhile to study other modifications and to attempt for synthesizing various substitutions on the piperidinyl nitrogen and their influence of homologuous series on the cytotoxic and CDK2 inhibitory activity.

Further, in a routine reaction set up, the cyclization of 2′ hydroxy chalcones by Algar Flynn Oyamada method (AFO) often results in the formation of Flavon-3-ols. However, it has its own limitation. In case when chalcones bearing methoxy groups on the ring A are subjected for AFO method, then, it could result in the formation of an aurone rather than flavonol. This could be presumably because of the alpha attack. This mechanism has been reported in detail by Nigam et al. (Nigam et al. 2017) and thus supported our study for the synthesis of aurones. Based on this, it was thought to synthesize novel substituted aurones.

## Methodology

### Molecular docking studies

The docking study was performed with maestro molecular modeling platform (version 10.5) available with Schrödinger LLC. The 3D X-ray crystallographic structure of CDK2/Cyclin A complexed with an oxindole inhibitor was downloaded from the protein data bank with a PDB id of 4FX3.The resolution for this protein structure is 2.75 Å units.

#### Step wise docking protocol

#### Protein preparation

The ligand-bound three-dimensional X-ray structure was obtained from the (Protein Data Bank [PDB] ID: 4FX3) with high resolution. The protein preparation was done by protein preparation wizard application. This includes major steps like preparation and refinement.

#### Ligand preparation

The ligand preparation was done using ligprep tool [Schrödinger release 2017]. The force field OPLS 2005 was used for this purpose. The ionization states were generated using the application EPIK. In this study, a series of 27 various piperidinyl substituted derivatives were considered in the docking process.

#### Ligand alignment

Flexible ligand alignment was carried out for obtaining better docking poses.

#### Extra precision Glide™ docking (Friesner et al. 2006)

GLIDE docking protocol involves two steps. In the first step, a GRID is generated and in the second step the designed ligands are docked into the active site and scored according to their binding free energy. The XP GLIDE uses a specific algorithm, that semi-quantitatively ranks the ligands by their ability to bind to a specific conformation of a protein receptor. The rigid receptor approximation used in the GLIDE supported us to evaluate ligands with maximum steric clashes, that otherwise would bind to a different conformation of the same receptor.

#### Validation of docking process

The RMSD (root mean square deviation) parameter was used to assess the accuracy of the docking protocol. In this case, RMSD was found to be 0.3, and was well within the limit of 2.

### MMGBSA- free binding energy calculations

The prime module of a commercial software Maestro 11.4 version (Schrodinger Inc.) was used to calculate the ligand binding and ligand strain energies of eight XP docked compounds using a Pose viewer file created in GLIDE XP mode. The VSGB solvation model, which uses the variable-dielectric generalized Born model and water as a solvent, and the OPLS3e force field to refine binding energy calculations, were used by Prime MM- GBSA.

### Molecular dynamics simulation

The molecular dynamics simulations were run using DESMOND tool available with the Schrodinger software. The protein ligand complex was preprocessed using protein preparation wizard option. The complex optimization and energy minimization was carried out during this step. The molecular dynamics system was constructed using system builder tool available with DESMOND package. The solvent model chosen was TIP3P with an orthorhombic box with a transferable intermolecular interactions of three points. OPLS 2005 force field was used in the present simulation. Counter ions were incorporated to create a neutral model. 0.1 M sodium chloride was added to mimic the normal physiological conditions. The NTP ensemble with a temperature of 300 K and 1 atmospheric pressure was chosen for the entire simulation. The MD trajectories were saved and analyzed for every 100 Pico seconds and their stability was assessed by determining root mean square deviation (RMSD) of the protein and ligand with the evolution of time. In the present simulation 70 nano second molecular dynamics simulation was run.

### Chemistry

All the reagents used in this study were procured from TCI or Merck Company with no further purification. ^1^H NMR and ^13^C NMR spectra were afforded by a Bruker Avance II spectrophotometer using CDCl3, DMSO as solvent and tetramethylsilane, as an internal standard. Chemical shifts were reported in parts per millions (ppm). All mass spectra were obtained using Waters LCMS systems. Melting points were also taken using an Electro thermal 9100 apparatus and were not corrected afterward. A Shimadzu spectrometer was utilized to record infrared spectra and the absorptions were expressed on the wave number (cm^–1^) scale ranged from 400 to 4000 cm^–1^.

#### General route for the synthesis of 1,2,3,6-tetrahydro-4-(2,4,6-trimethoxyphenyl)-1-methylpyridine,1,2,3,6-tetrahydro-4-(2,4,6-trimethoxyphenyl)-1-ethylpyridine,1,2,3,6tetrahydro-4-(2,4,6-trimethoxyphenyl)-1- propyl pyridine,1,2,3,6-tetrahydro-4-(2,4,6-trimethoxyphenyl)-1-iso propyl pyridine (Liu and Go 2006)

Equimolar quantity (0.005 mol) of the corresponding N-alkyl piperidone (methyl, ethyl, propyl, isopropyl) was added with constant stirring to a solution containing 0.84 g of 1,3,5- trimethoxy benzene in glacial acetic acid under inert (calcium guard tube) atmosphere, while maintaining the temperature at 20 °C. To the resulting dark brown solution, 10 ml of concentrated HCl was added. Alternatively, HCl gas was bubbled at a rapid rate through this mixture for 1–2 h. After stirring for 7–8 h, the reaction mixture got transformed itself into a dark purple solution. It was then subjected to constant heating on a sand bath maintained at 80–90 °C for 7–8 h. The solvent was removed under pressure. To the concentrate, crushed ice was added followed by the addition of cold distilled water. Further, the cold solution was filtered to remove trace amount of unreacted trimethoxy benzene (approximately equivalent to 0.0002 mol). To the resulting filtrate, 150 g ice was added and the pH was adjusted to 10–11 by the addition of 50%w/v NaOH aqueous solution dropwise, while maintaining conditions below 5 °C. The white precipitate separated out from the solution was filtered and washed repeatedly with ice cold water.

Further, the product obtained was recrystallized with acetone: water (2:1) mixture to obtain pale pink needle shaped crystals of intermediate (**I**). The melting point was measured using Toshniwal melting point apparatus and were uncorrected.

#### General procedure for the synthesis of 1-[3-(1-Methyl-1,2,3,6-tetrahydro-pyridin-4-yl) 2 hydroxy-4,6-dimethoxy-phenyl]-ethanone, 1-[3-(1-Ethyl-1,2,3,6-tetrahydro-pyridin-4-yl)-2- hydroxy-4,6-dimethoxy-phenyl]-ethanone, 1-[3-(1-Propyl-1,2,3,6-tetrahydro-pyridin-4-yl)-2- hydroxy-4,6-dimethoxy-phenyl]-ethanone, 1-[3-(1-Isopropyl-1,2,3,6-tetrahydro-pyridin-4-yl)-2- hydroxy-4,6-dimethoxy-phenyl]-ethanone (II)

To an ice cold solution of 1.2 g of the intermediate-(**I**) (0.003 mol) in 15–20 ml of dichloromethane, 4.2 ml of boron trifluride diethyl etherate (0.065 mol) was added drop wise with constant stirring under anhydrous reaction conditions. After 6–8 h of constant stirring on an ice bath at a temperature of 0–5° C, the reaction mixture turned into a buff colored solution. To this, 4.8 ml of acetic anhydride (0.049 mol) was added drop wise. The solution was then stirred for 48 h in the dark at room temperature under anhydrous conditions. After 48 h, the reaction mixture was concentrated by removing dichloromethane and further poured into crushed ice. It was then neutralized with 20%w/v Na2CO3 aqueous solution. The brown solid precipitated out was washed with water, filtered and dried. The dried crude product was purified by recrystallization using methanol: water mixture (2:1) to obtain yellow colored needle shaped pure intermediate (**II**). The melting point was measured using Toshniwal melting point apparatus and were uncorrected.

#### General procedure for the synthesis of 1-[3-(n-alkyl-1, 2, 3, 6-tetrahydro-pyridin-4-yl)-2 hydroxy-4, 6-dimethoxy-phenyl]-3-(substituted phenyl)-propenone or chalcones: (Liu and Go 2006)

Further, the purified intermediate-(**II**), 0.367 g (0.001 mol) was dissolved in 5 ml of 20% w/v KOH in ethanol solution. To this solution, various substituted aldehyde (0.0012 mol) was added and the reaction mixture was stirred at room temperature under anhydrous conditions. After the completion of the reaction, the mixture was poured into crushed ice drop wise with constant stirring and the precipitate so obtained was filtered and washed several times with cold water. The crude product obtained was then purified by recrystallization using different mixtures of organic-aqueous solutions. Further, purification was done by column chromatography using silica gel (100–200 mesh) packed column with chloroform: methanol as mobile phase (gradient elution) upto 1.5% v/v methanol to obtain pure products. The melting point of all the test compounds were measured using Toshniwal melting point apparatus and were uncorrected.

#### General procedure for the synthesis of 7-(1-alkyl-1, 2, 3, 6-tetrahydro-pyridin-4-yl)-2- (substituted-benzylidene)-4,6-dimethoxy-benzofuran-3-one or aurones. (Narsinghani et al. 2013)

Substituted chalcone (0.001 mol) was dissolved in 20 ml of 20% w/v KOH in methanol solution till a clear solution was obtained and maintained on an ice bath at 5–10 °C with constant stirring. To this solution, 2 ml of 30% w/w hydrogen peroxide (H2O2) solution was added dropwise and the mixture was then continuously stirred for 6–8 h while maintaining the reaction condition at 15–20 °C in a closed reaction vessel. After the completion of the reaction, the precipitate was filtered and washed several times with cold water. The crude product so obtained was then subjected to purification by column chromatography on silica gel (100–200 mesh) using chloroform: methanol as mobile phase following gradient elution technique upto 1% v/v of methanol. Further, the product obtained from column chromatography was recrystallized using solvent mixtures consisting of different organic and aqueous solvents to obtain the final compounds. The melting point of all the test compounds was measured using Toshniwal melting point apparatus and were uncorrected.

All the spectral characterisation data (UV, IR, MASS and NMR ^1^H, ^13^C) were provided in supplementary data.

### Anti-oxidant evaluation

#### DPPH. free radical scavenging assay

DPPH. assay was performed using 96-well plate by serial dilution method. The dilution of methanolic stock solution of the test compounds and standard anti-oxidants was carried out to obtain concentration in the range of 3.9–1000 µM. Quercetin and ascorbic acid were used as standards. To a 100 μL of this solution, 100 μL of 200 µM DPPH^.^ in methanolic solution was added. The experiment was done in triplicate and the plate was then incubated for 30 min in the dark and later the absorbance was measured at 517 nm using Elisa reader. The percentage DPPH^.^ Scavenging was calculated using the formula, (Control Absorbance–Test Absorbance)*100/(Control Absorbance). The anti-oxidant activity of the test compounds was expressed in terms of concentration required for 50 percentage inhibition (IC_50_) (Collins 2005).

#### ABTS. + radical scavenging assay

The assay was performed in a 96-well plate by serial dilution method. A 2 mM ABTS^.+^ working solution was prepared in a phosphate buffer (Zheleva-Dimitrova et al 2010).To this, 200 μL of aqueous potassium per sulfate (70 mM) solution was added and the solution was incubated for 24 h in the dark at room temperature to develop a blue-green color. To 30 μL of ABTS^.+^ working solution, 50 μL of different concentrations (3.9–1000 μM) of the test sample solution and 170 μL of phosphate buffer (pH-7.4) were added in a well. The plate was then incubated in the dark at room temperature for 10 min. Then absorbance was measured at 690 nm using Elisa reader. Quercetin and ascorbic acid were used as standards. The percentage scavenging of ABTS^.+^ was calculated using the formula, (Control Absorbance–Test Absorbance)*100/(Control Absorbance) and the anti-oxidant activity was expressed in terms of IC50 value.

### Anti-cancer activity (MTT assay) on MCF-7 and HCT-116 cell lines

The monolayer cell culture was trypsinized and the cell count was adjusted to 5.0 × 10^5^ cells/ml using respective media containing 10% FBS. To each well of the 96-well microtiter plate, 100 µL of the diluted cell suspension (50,000cells/well) was added. After 24 h, when a partial monolayer was formed, the supernatant was flicked off, the monolayer was then washed once again with the medium and 100 µL of different test concentrations of test drugs were added on to the partial monolayer in microtiter plates. The plates were then incubated at 37 °C for 24 h in 5% CO_2_ atmosphere. After incubation, the test solutions in the wells were discarded and 100 µL of MTT (5 mg/10 ml of MTT in PBS) was added to each well. The plates were then incubated for 4 h at 37 °C in 5% CO_2_ atmosphere. The supernatant was then removed and 100 µL of DMSO was added and the plates were gently shaken to solubilize the formed formazan. The absorbance was measured using a microtiter plate reader at a wavelength of 590 nm. The percentage growth inhibition was thus calculated using the following formula and the concentration of the test drug required to inhibit the cell growth by 50% (IC_50_) values is generated from the dose–response curves for each one of the cell lines (Meerloo et al. 2011).

### Assessment of DNA damage by comet assay

#### Procedure for the comet assay

To assess the genotoxicity effect of newly synthesized aurones, comet assay was performed on HCT -116 cell lines.

#### Preparation of slides

The agarose-coated glass slides were prepared according to the standard procedure (Tice et al. 2000).

Compound selected—NISOA4.

Cell line used were- HCT116 cells (Human colorectal cancer cell lines).

HCT -116 (1 × 10^6^) cells were treated with two different concentrations, 12.5, 25 μM of NISOA4 for 24 h.

#### Isolation of cells

The cells were centrifuged at 2000 rpm at 4 °C for 5 min followed by washing with PBS. Further, it was air dried and pellet was dissolved once again in PBS.

#### Electrophoresis under pH > 13 in alkaline condition and neutralization of microgel slides

After keeping slides at 4 °C for 2 h, slides were removed from the lysis buffer and were placed side by side on a horizontal gel box near one end, sliding them as close together as possible.

The slides were placed on ice for 10 min and submerged in lysis buffer (2.5% NaCl, 100 mM EDTA, 10 mM Tris, 10% DMSO and 1% Troton X-100) at pH 10 at 4 °C for more than 1 h.

In the next step, the buffer reservoir was filled with freshly made electrophoresis buffer (pH > 13) until the liquid level was enough to cover the slides. Later, the slides were dipped in alkaline buffer (30 mM NaOH, 1 mM EDTA) for about 20 min so as to allow for unwinding of the DNA and the expression of alkali-labile damage. Further, the power supply was turned on and adjusted to 18–24 Volts (~ 0.74 V/cm) and current of 300 mA by raising or lowering the buffer level. The electrophoresis was then run for 30 min. Later, the slides were gently removed and washed repeatedly with distilled water followed by 70% chilled ethanol. They were finally air dried and further taken for staining procedure.

#### Staining

Slides were stained with 80 µL 1× Ethidium Bromide and left aside for 10 min followed by washing with PBS to remove the excess of stain. The slides were then scored immediately after washing.

#### Evaluation of DNA damage

The visualization of DNA damage was observed based on EtBr-staining technique using a 40× objective on a fluorescent microscope. The assessment of both qualitative as well as quantitative extent of DNA damage in the cells were carried out by measuring the length of DNA migration and the percentage of the migrated DNA using ImageJ software with open comet plug-in. Generally, 50 to 100 randomly selected cells are analysed per sample. Finally, the total tail amount was calculated. The final results are assessed by using the following parameters: (i)amount of migration per cell (ii) the number of cells with increased migration (iii) the extent of migration among damaged cells and (iv) cell viability (Końca et al. 2003).

### Apoptotic DNA fragmentation studies

Cells were seeded at a concentration of 1 × 10^6^ per 35 mm dish and incubated at 37 °C/5% CO_2_ for 24 h. The confluent cells grown after 24 h of incubation were treated with sample concentrations at 10 μg/ml, 20 μg/ml and control (without sample). After the treatment, cells were trypsinized and both the adherent as well as floating cells were collected by centrifugation at 2000 rpm for 5 min. The cell pellet was later suspended in 0.5 ml lysis buffer (pH 7.8) [Tris–HCl 10 mM, pH 8; EDTA 20 mM, pH 8.0; TritonX-100 0.2% and 4 M NaCl], vortexed vigorously and incubated at 50 °C for 5 min. To the lysate, 0.5 ml of phenol: chloroform: isoamylalcohol was added and mixed for about 2–3 min. Further, it was centrifuged at 10,000 rpm for 15 min at 4 °C.The upper aqueous layer was taken in another tube to which, twice the volume of cold 100% ethanol was added along with 3 M sodium acetate (Final concentration of sodium acetate 0.3 M). It was incubated for 5–10 min at room temperature. Further, centrifuged again at 10,000 rpm for 20 min. After removing the supernatant, the DNA pellet was washed in 70% ethanol and centrifuged at 5000 rpm for the next 10 min. The supernatant was removed, the DNA pellet was air dried and was finally dissolved in TE buffer (Tris–HCl 10 mM, pH 7.4, EDTA 1 mM, pH 8.0) and separated by 2% agarose gel electrophoresis at 100 V for 50 min.

### Cell cycle analysis

The cell cycle analysis was followed where 1 × 10^6^ cells were seeded and cultured for 24 h in a 6-well plate containing 2 ml of the media. Cells were then treated with desired concentrations of the given samples that were prepared in media and further incubated for another 24 h. Cells were then harvested and centrifuged at 2000 rpm for 5 min at room temperature and the supernatant was discarded carefully retaining the cell pellet. Cell pellet was then washed by resuspending in 2 ml of 1XPBS. The washing was repeated another time under the same conditions. Further, the supernatant was discarded retaining the pellet. Cells were then fixed by resuspending in 300 µL of Sheath fluid followed by the addition of 1 ml of chilled 70% ethanol drop by drop gently with continuous shaking and one more mL of the same at once. The cells were then stored at 4 °C overnight. Post fixing, the cells were centrifuged at 2000 rpm for 5 min washed twice with 2 ml of cold 1XPBS and suspended in 450 µL of sheath fluid containing 0.05 mg/ml PI (Propidium iodide), 0.05 mg/ml RNase A and incubated for 15 min in the dark (Kasibhatla et al. 2006).The percentage of cells in various stages of cell cycle both in the treated as well as un-treated populations was determined using FACS Caliber (BD Biosciences, San Jose, CA).

#### Apoptosis study using propidium iodide and ANNEXIN V-FITC method

The day before induction of apoptosis, 1 × 10^6^cells per well were plated on a 6-well plate using respective media with 10% FBS and 1% PenStrep, incubated overnight at 37 °C at 5% CO_2_.The media was replaced with test solutions of different concentrations in the media containing 10% FBS. The treated cells were incubated for 24 h at normal culture conditions. The cells were further harvested and the well contents were completely transferred into the sterile FACS tubes. The cell contents were centrifuged at 2000 rpm for 5 min and the supernatant was discarded. After centrifugation, the cells were washed twice with cold PBS and then resuspended in 1 ml of binding buffer at a concentration of 1× 106 cells/ml. 500 mL of the cell suspension (5× 105 cells) were then transferred into a fresh FACS tube (Vermes et al. 1995). Further, cells were gently mixed with 10 mL of propidium iodide and 5 mL of Annexin V before being incubated for 20 minutes at room temperature in the dark. The cells were immediately analyzed by flow cytometer within a duration of an hour.

### Semi -quantitative RT-PCR (reverse transcriptase) based gene expression studies on the target gene encoding CDK2/cyclin A

#### Sample preparation and RNA isolation (Chomczynski and Sacchi 1987)

HCT-116 cells were trypsinized and added to 1 ml TRIzol and vortexed. Samples were then allowed to stand for 5 min at room temperature. 0.2 ml of chloroform per 1 ml of TRIzol was added. The tube was later stoppered and shaken vigorously for 15 s and was allowed to stand at room temperature for 5 min. The resulting mixture was then centrifuged at 10,000×rpm for 15 min at 40 °C. The upper aqueous phase was transferred to another clean tube followed by the addition of 0.5 ml of isopropanol per 1 ml of TRIzol. The contents were mixed gently by inverting the sample 5 times and incubated at room temperature for 5 min. Further, the tubes were centrifuged at 10,000×rpm for the next 10 min at 40 °C. Later, the supernatant was discarded and the RNA pellet was washed by adding 1 ml of 70% ethanol. Further, the contents were mixed gently by inverting the sample for few times. It was again centrifuged for 5 min at 14,000×rpm at 40 °C. In the later step, the supernatant was discarded by inverting the tube on a clean tissue paper. Later, the pellet was dried by incubating in a dry ice bath for 5 min at 55 °C. The pellet was then resuspended in 25 µL of DEPC (Diethyl pyro carbonate) treated water.

#### RT-PCR (reverse transcriptase polymerase chain reaction)

A semi-quantitative reverse transcriptase polymerase chain reaction (RT-PCR) was carried out using Techno Prime system to determine the levels of β-actin and CDK-2 and CyclinA mRNA expression. The cDNA was synthesized from 2 µg of RNA using the Verso cDNA synthesis kit (Thermo Fischer Scientific) with oligo dT (deoxy thymine) primer according to the manufacturer’s instructions. The reaction volume was set to 20 μL and the cDNA synthesis was performed at 42 °C for 60 min followed by RT inactivation at 85 °C for 5 min (Murakami 1996).

#### PCR (polymerase chain reaction)

The PCR mixture (final volume of 20 µL) contains 1 µL of cDNA, 10 µL of Red Taq Master Mix 2× (Ampliqon) and 1 µM of each complementary primer specific for CDK2 and CyclinA and β-actin (internal control) sequence. The samples are denatured at 95 °C for 5 min and amplified using 35 cycles of 95 °C for 30 s then again at 55 °C for 30 s and 72 °C for 1 min for β-actin; the p53 annealing was finally set to 56 °C followed by the final elongation at 72 °C for 10 min. The optimal numbers of cycles used for amplification of these two genes experimentally are selected in such a way that the final amplifications are in the exponential range and do not reached a plateau. Ten microliters of the final amplification product are run on a 2% ethidium-stained agarose gel and photographed. Quantification of the results could be accomplished by measuring the optical density of the bands, using the computerized imaging program Image J. The values were normalized to β-actin intensity levels (Bookout and Mangelsdorf 2003). The primers procured were shown in Table 1.

**Table 1:** Primers synthesized at Eurofins Genomics, India

Gene	Primer pair	Sequence	Tm	Product size (bp)
β-actin	FP	TCCTCCTGAGCGCAAGTACTCT	52	153
	RP	GCTCAGTAACAGTCCGCCTAGAA		
CDK-2	FP	CCAGTGAAAATGAAAGGAAG	45	138
	RP	AAATCTAACGTGTAGGAAGG		
Cyclin A	FP	AAACAAGTTCTGAGAATGGA	43	146
	RP	CCCAAAAACATTGCTAAACT		

### CDK2/cyclin A inhibitory assay using FRET (florescence resonance energy transfer) analysis

#### Assay principle

Z’-LYTE^®^ technology involves homogeneous assay design based on FRET and is a universal platform for both screening as well as profiling protein kinases. The assay uses a peptide substrate labeled with two different fluorophores, coumarin donor and fluorescein acceptor on each end. These two fluorophores make up a FRET pair, which allows the transfer of energy when both are present on the same molecule. The initial kinase reaction transfers the ϒ-phosphate of ATP to a tyrosine or serine/threonine residue on the substrate. In a secondary reaction, referred to as the development reaction, a site-specific protease recognizes and cleaves only non-phosphorylated peptide, while phosphorylated substrate remains uncleaved. Uncleaved phosphorylated product would exhibit FRET emission, whereas cleaved peptides would not. Upon excitation of Coumarin at 400 nm, the FRET signal is measured as a ratio between the Coumarin donor emission at 445–460 nm and the fluorescein acceptor emission at 520–535 nm(Lebakken et al. 2007).

#### Assay procedure

#### Optimization of kinase, ATP concentrations

Initial ten-fold and two-fold dilutions of Kinase and ATP concentrations were used to choose the optimal Kinase and ATP concentration based on the % Phosphorylation. The results suggested that the maximal activity of kinase and ATP was found to be at 1000 ng/ml Kinase concentration and 1000 µM ATP with phosphorylation of 39.77%.

The assay was carried out according to the kit instructions (384 well format). Briefly, 5X kinase buffer [250 mM HEPES (pH 7.5), 50 mM MgCl2, 5 mM EGTA, 0.05% BRIJ-35] was diluted to 1X using distilled water. The phosphor peptide positive control (Z’-LYTE^®^ Tyr 4 phospho-peptide) and the non-phosphorylated negative control (Z’-LYTE^®^ Tyr 4 peptide) were then diluted to 1:500 with 1× kinase buffer to 2 μM. A series of mixtures, each with 2 μM peptide ranging from 100% phosphor peptide were brought to 0%, then were made by combining various amounts of phosphorylated and non-phosphorylated peptides to mimic the results of a kinase reaction. Working development solution was then made by diluting 12 μL of the provided stock solution with 388 μL of the provided development buffer. Aliquots (20 μL) of each peptide mixture were then added to the wells of a Corning 3914 solid white microplate in replicates of 8. The development reaction was initiated by the addition of 10 μL of working development-solution to all the wells. Further, the plates were allowed to incubate at room temperature for 60 min. Reactions were then stopped by the addition of 10 μL of stop solution (Jia et al. 2008).

Reactions were quantitated using a Tecan Robotic Microplate Reader. Reader control, blank subtraction, ratio metric calculations and finally graph plotting were carried out using Gen5™ Data Analysis Software.

## Results and discussion

### Molecular docking studies

**Fig. 3:**
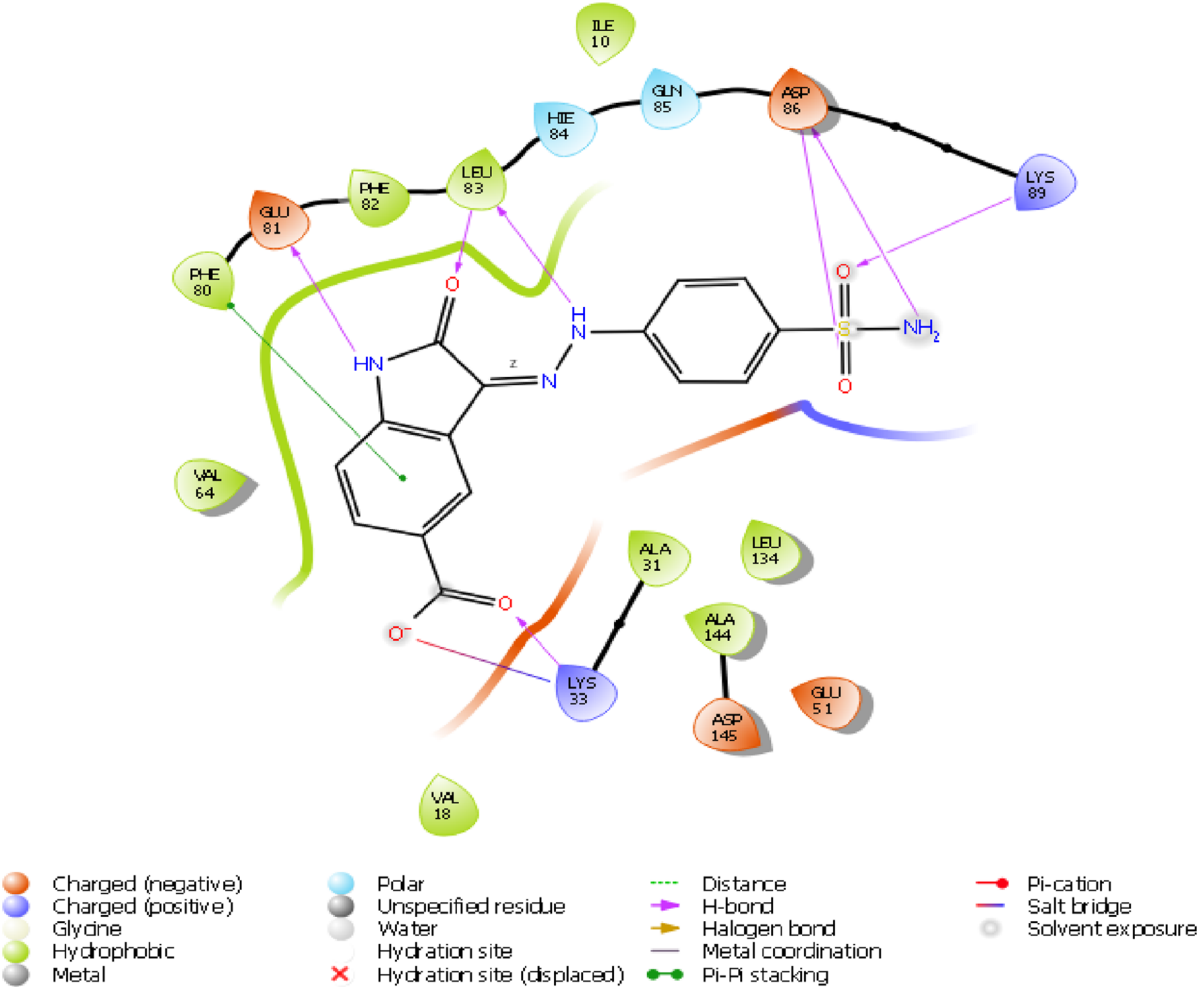
The 2D interaction of Crystal ligand at the active site of CDK2/cyclin A

**Fig. 4:**
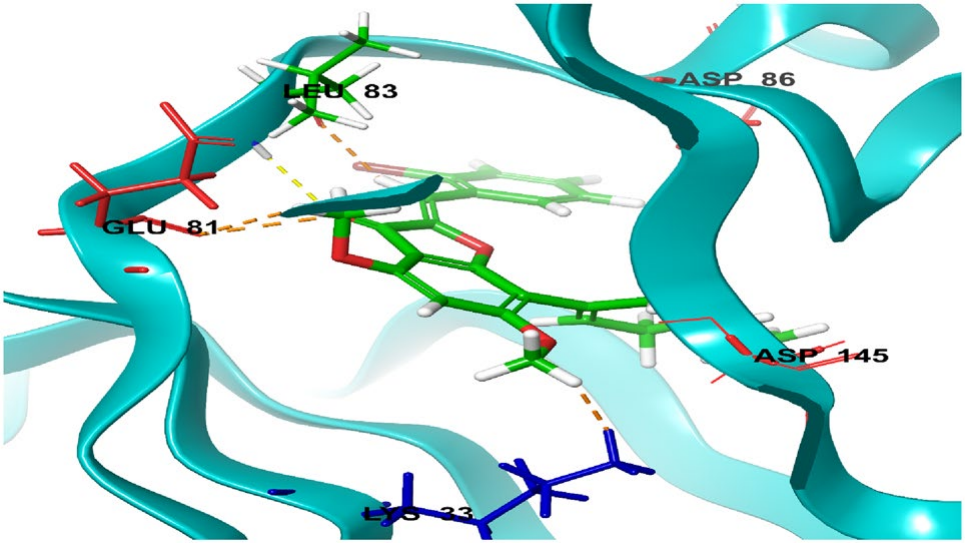
Active compound NMA2 at the active site of CDK2/cyclin A

**Fig. 5:**
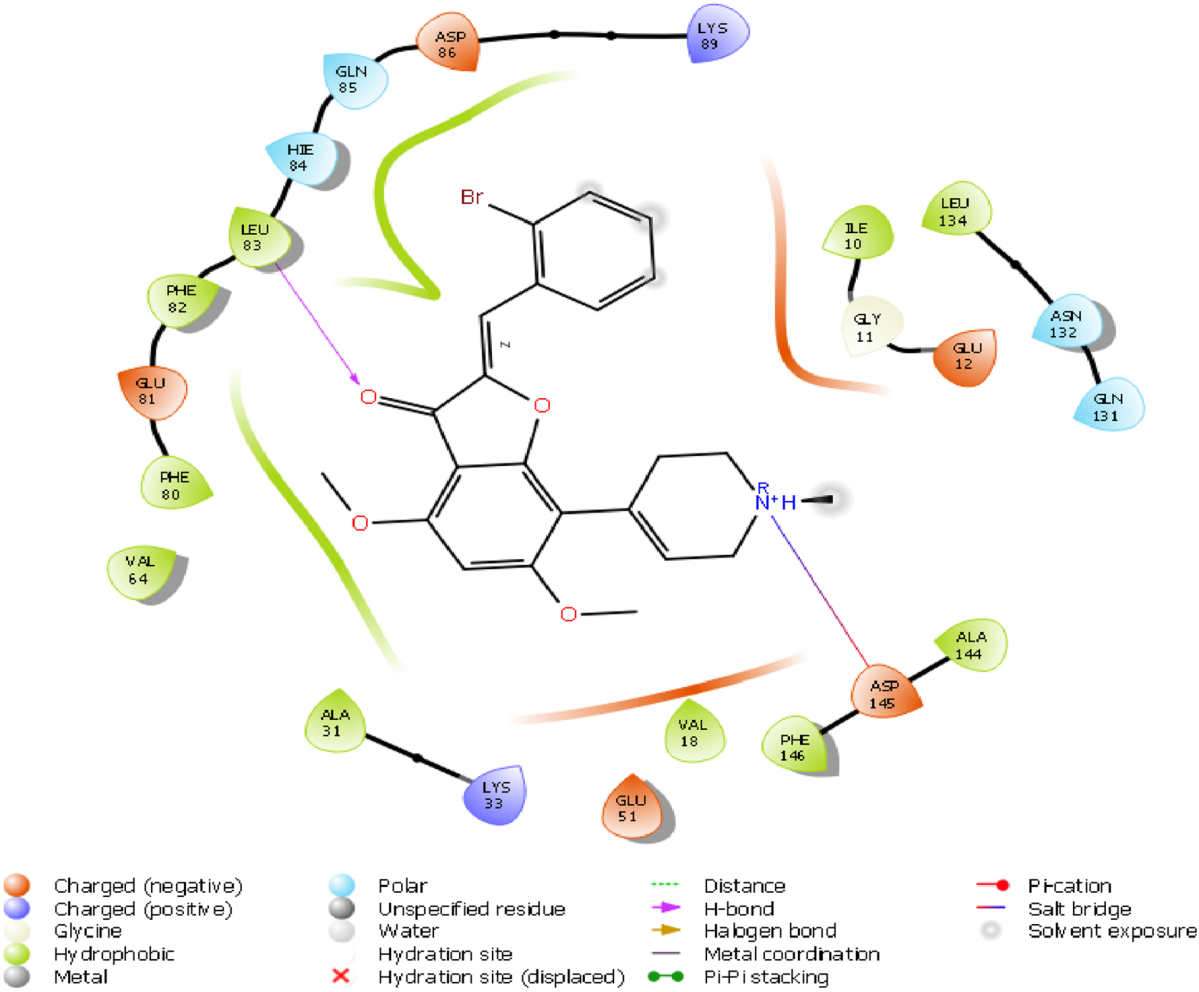
The 2D interaction of NMA2 at the active site of CDK2/Cyclin A

Structural information based on the crystal structure of CDK2/cyclin A complexed with oxindole inhibitor was presently used to study the active site interactions as shown in Fig. 2. This scaffold occupies the ATP pocket of the most stable conformer of the kinase. The compound makes significant interaction with the possible active site amino acids. It is worthwhile to note that the nitrogen of the oxindole ring tend to form a hydrogen bond donor interactions with the amino acid Glutamine 83; however, the nitrogen of the hydrazine moiety forms a hydrogen bond donor interaction with the amino acid leucine 83.Further, the side chain hydroxyl and amino groups forms a hydrogen bond donor interactions with the amino acid aspartic acid 86. The most active aurone in the series NMA2 forms hydrogen bond donor interactions with aspartic acid 145 and hydrogen bond acceptor interactions with the amino acid leucine 83. Crystal ligand 2D and 3D interactions at the active site is represented in Figs. 2 and 3. Interactions of the most active aurone NMA2 is represented in Figs. 4 and 5. The docking scores for all the aurones is represented in Table 2. The amino acid interactions of crystal ligand, flavopiridol and high dock score compounds are presented in Table 3.

**Table 2:** Docking scores of designed Aurones

ligand	GScore	DockScore
4FX3—hbond-opt_ligand	− 12.81	− 12.81
Flavopiridol	− 9.41	− 9.37
NMA2	− 9.05	− 9.04
NPA2	− 9.02	− 9.01
NMA3	− 9.02	− 9
NISOA4	− 8.91	− 8.89
NEA1	− 8.82	− 8.81
NMA1	− 8.72	− 8.71
NEA2	− 8.63	− 8.61
NEA3	− 8.59	− 8.58
NISOA3	− 8.59	− 8.58
NPA1	− 8.53	− 8.52
NISOA2	− 8.46	− 8.45
NEA6	− 8.43	− 8.42
NISOA6	− 8.11	− 8.1
NPA3	− 8.03	− 8.02
NEA4	− 7.84	− 7.83
NMA5	− 7.81	− 7.8
NMA6	− 7.64	− 7.63
NEA5	− 7.46	− 7.45
NPA4	− 7.24	− 7.23
NEA3-2	− 9.08	− 6.78
NISOA1	− 6.28	− 6.26
NISOA3-2	− 8.18	− 5.89
NMA4	− 7.89	− 5.6
NPA5	− 5.39	− 5.38
NISOA5	− 3.14	− 0.84

**Table 3:** The interactions of top scoring compounds with various amino acid residues

Name	Hydrophobic	Pi-Pi stacking	Pi-cationic	H-bond
Crystal Ligand	–	Phenyl alanine 80	–	Lysine 33 Aspartic acid 86 Lysine 89 Leucine 83 Glutamine 81
Flavopiridol	**–**	Phenyl alanine 80	**–**	Aspartic acid 145 Glutamine 81 Leucine 83
NMA2	**–**	**–**	**–**	Leucine 83 Aspartic acid 145
NPA2	**–**	**–**	**–**	Leucine 83
NMA3	**–**	**–**	**–**	Aspartic acid 145 Leucine 83

### MMGBSA- binding free energy calculations

Prime- MMGBSA tool was used for binding energy and strain energy prediction of a set of ligands involved in protein ligand complex. After molecular docking, the docked poses of 27 newly designed aurones were subjected to Prime-MMGBSA to determine the binding free energy, i.e., dGbind of their poses. The designed compounds such as NMA2, NEA3, NISOA1, NISOA1, NISOA2,NISOA3,NISOA4,NISOA5 and NISOA6 exhibited dG bind > − 50 kcal/mol indicating stable binding at the active site. In comparison to CDK2 percentage inhibition, the compound with higher percentage inhibition values also showed higher free energy of binding values suggesting a better correlation between CDK2 inhibitory activity and free energy of binding values, whereas comparison of docking scores with the percentage CDK2 inhibition values could not give any significant correlation.

### Molecular dynamics simulation

**Fig. 6:**
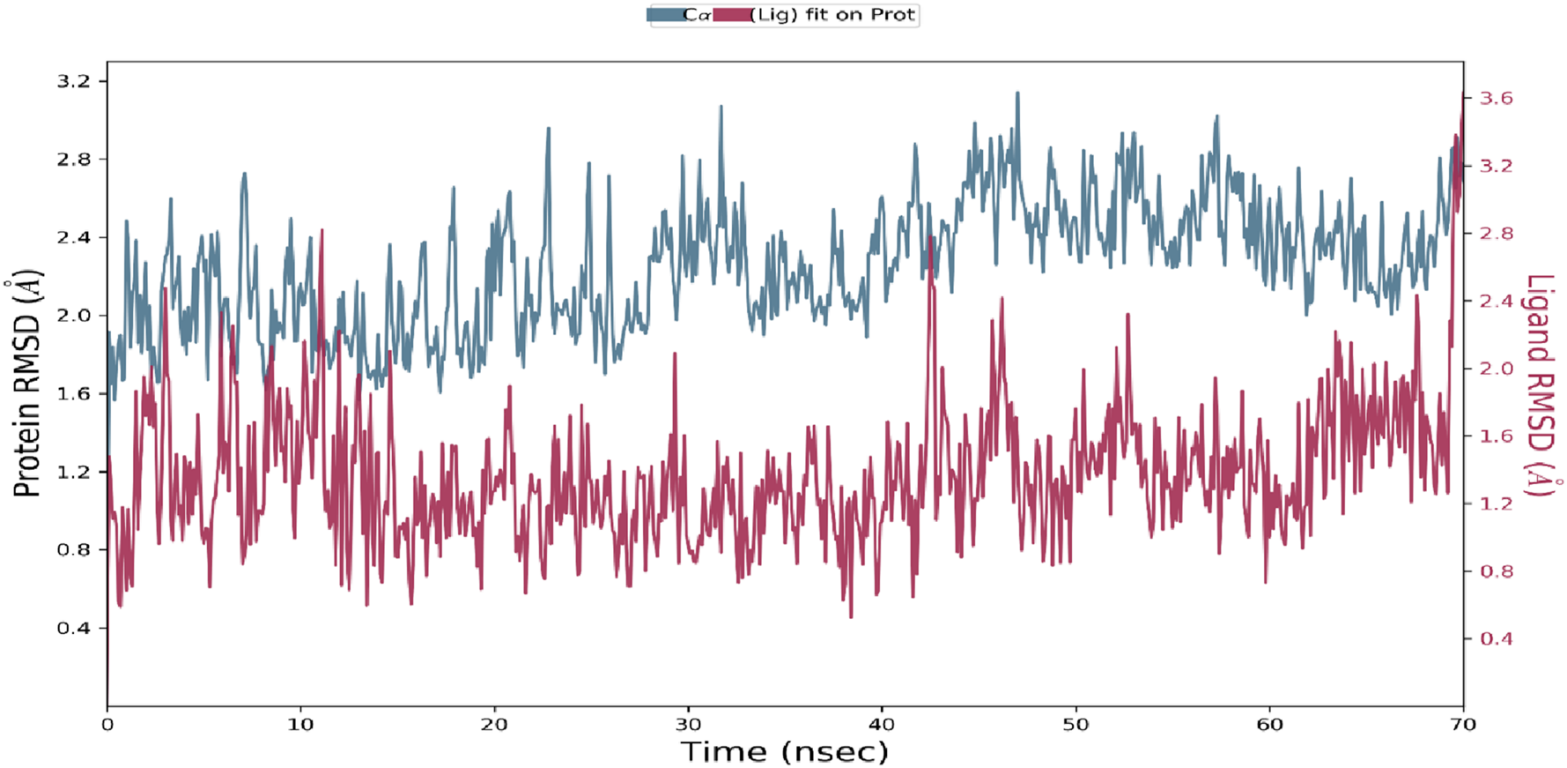
Root mean square deviation (RMSD) of c-alpha atoms of the protein with the time evolution. The left Y axis display the variation in the protein backbone with the time progression

**Fig. 7:**
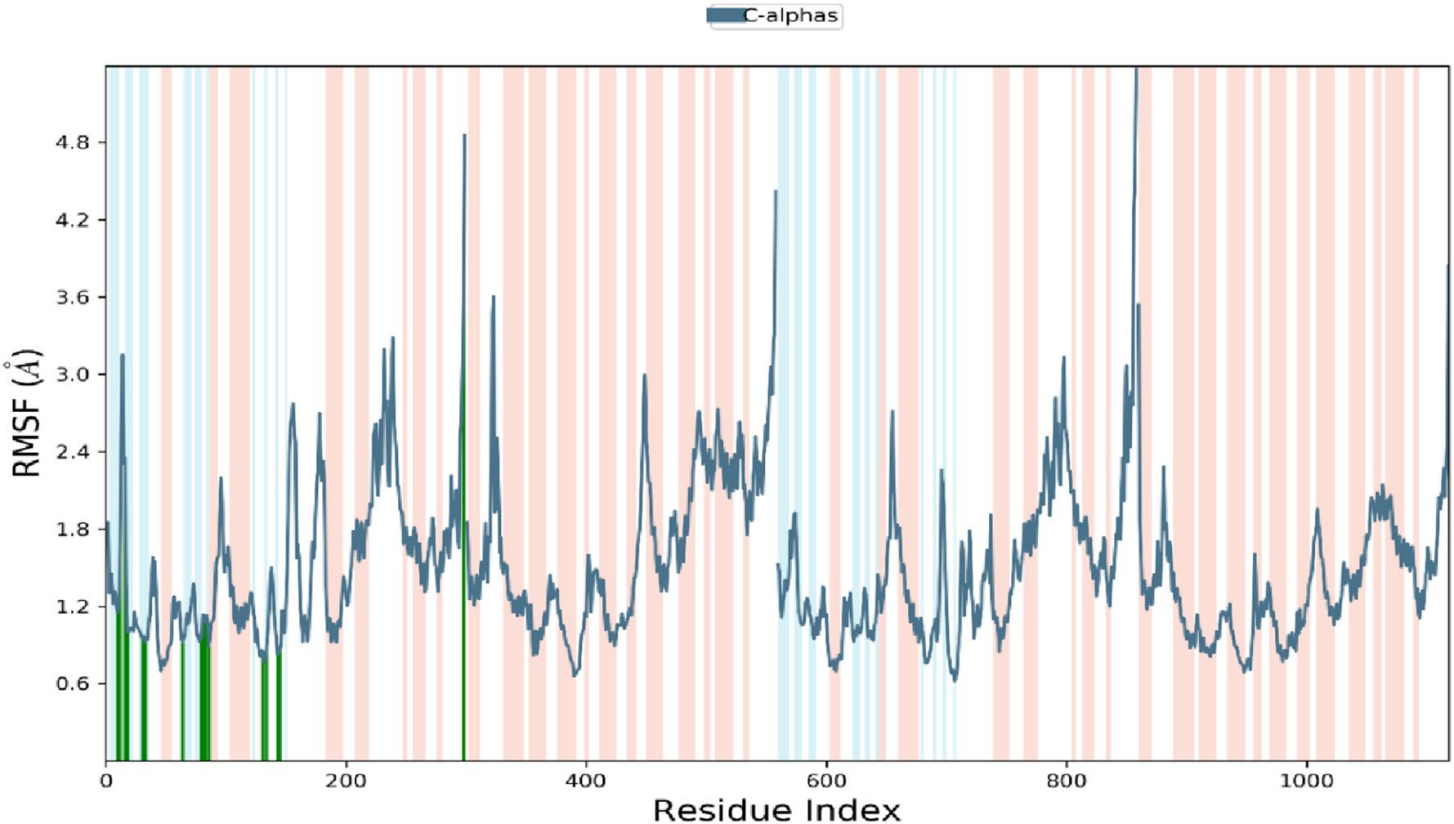
Root mean square fluctuation (RMSF) of the c-alpha atoms of the protein with the time increment. The left Y axis shows the variation of protein RMSF and X axis shows B-factor thought the simulation time. Ligand atoms interacting with the active site residues are marked with green colour

**Fig. 8:**
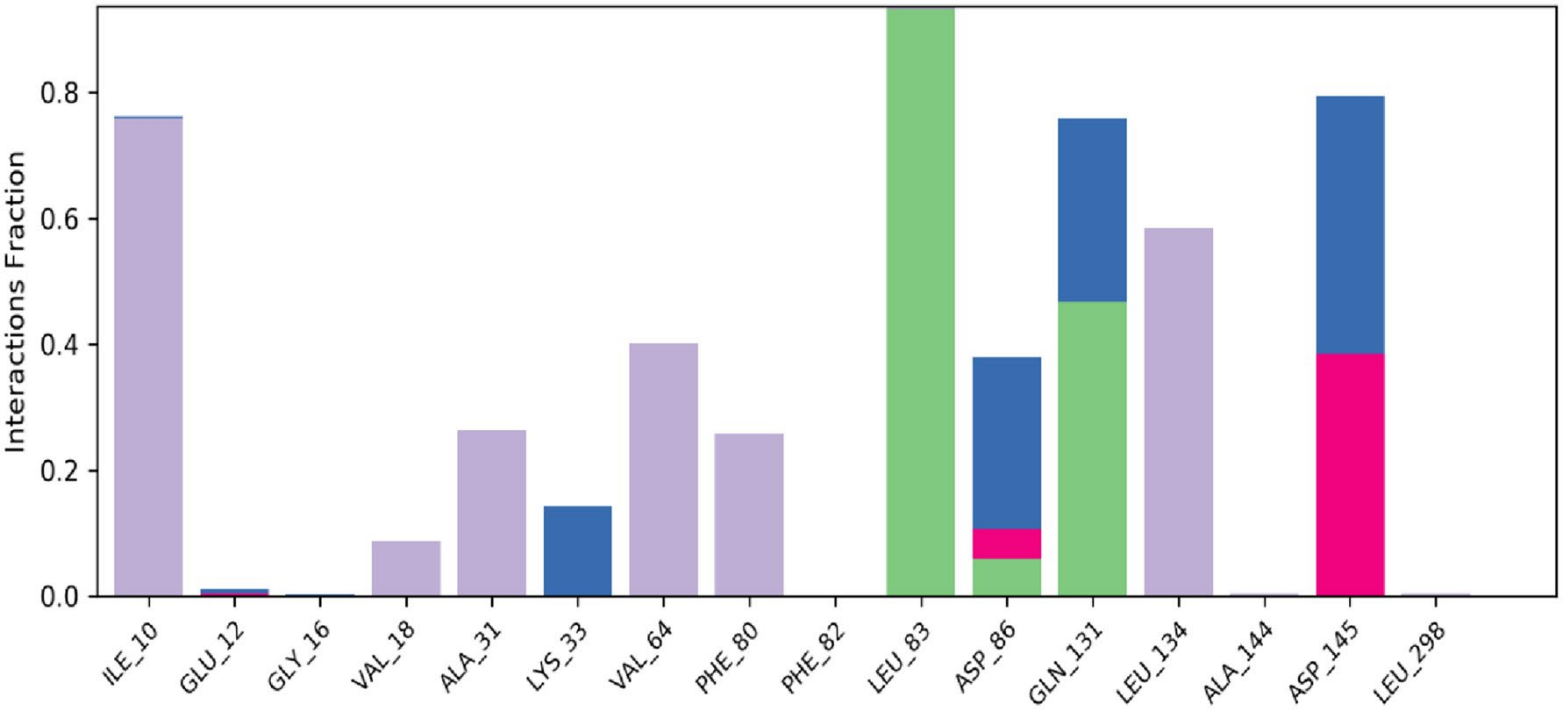
Protein ligand contact with percentage interactions

**Fig. 9:**
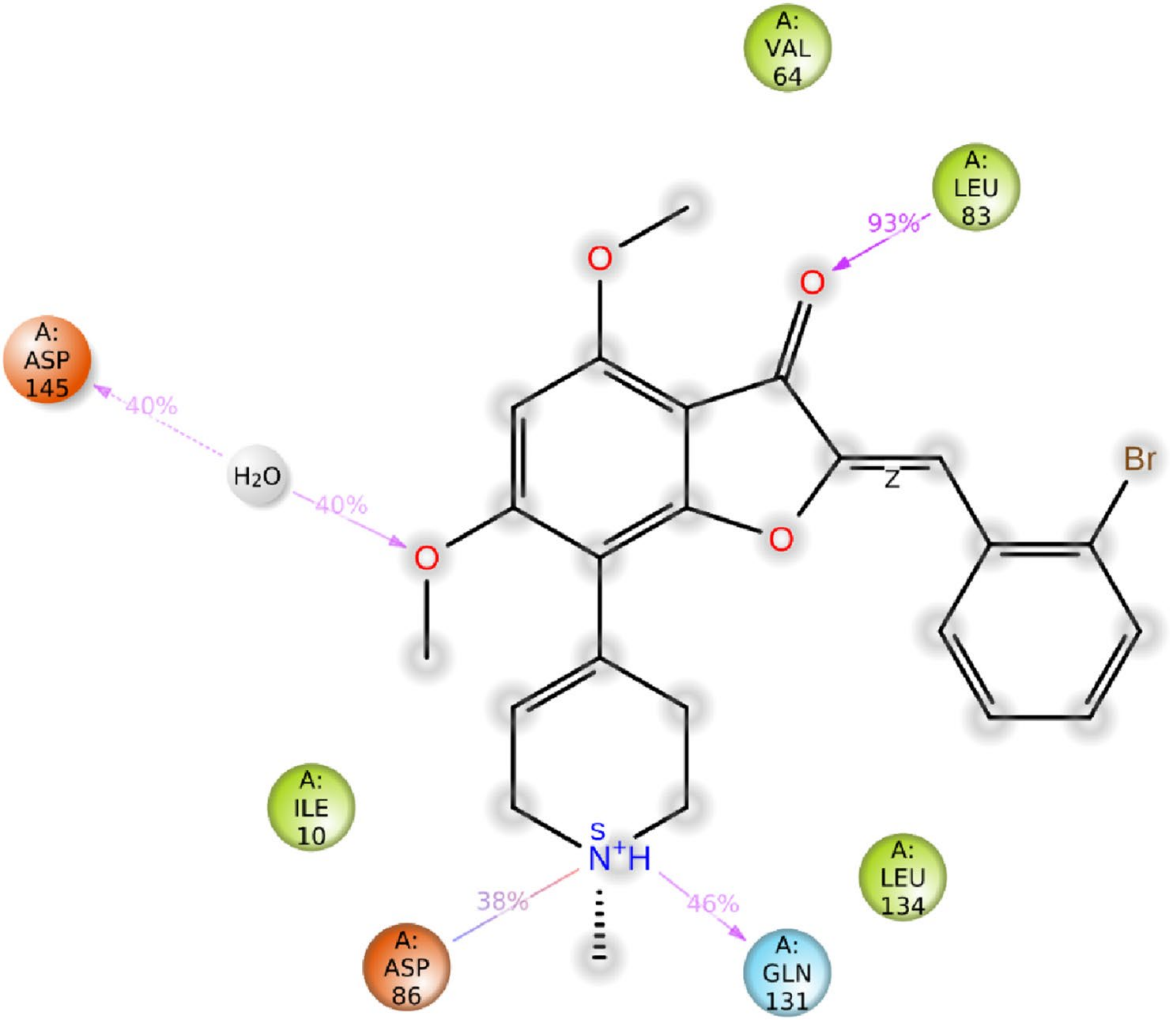
Ligand atom interactions with the protein residues

Molecular docking studies will only predict the ligand interacting at the active site of the receptor in static settings. Docking poses are not sufficient to predict the atom movements with time evolution. Molecular Dynamics simulations tend to calculate atomic movements with time by integrating Newton’s equation of motion. The ligand binding status at the physiological conditions were predicted using MD simulations. The protein ligand interactions, root mean square deviation (RMSD) and root mean square fluctuations (RMSF) were determined through MD trajectory analysis. As shown in the Figs. 6 and 7 root mean square deviation and root mean square fluctuation will provide powerful insights on protein complex equilibrium and fluctuations thought the simulation time. In figure RMSD results shows that CDK2-titled compound are converged lesser than 2 Å, which indicates the system has small conformation and the system is a small or a globular protein. The RMSD values were consistent and stable during the course of the simulation. In figure RMSF plot will help us to analyze the occurrence of local changes along with the protein chain and green coloured lines represent the binding of compound NMA2 with the active site amino acid residues of CDK2. Figures 8 and 9 show the final molecular dynamics output of CDK2-NMA2 complex. The most prominent ligand–protein interactions discovered by MD simulation were hydrogen bonding interactions. For complex residues ILE 10, LEU 83 and LEU 134 are the essential hydrogen bonding interactions. Individual ligand atom interactions with protein residues are presented in Fig. 9.

### Chemistry

**Fig. 10:**
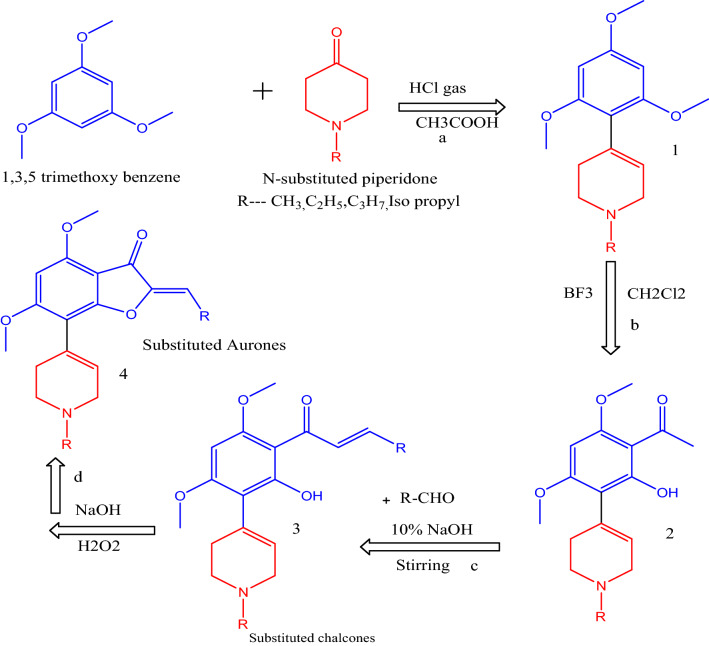
Scheme for the synthesis of intermediates and final compounds

According to Fig. 10 the synthesis of title compounds was carried out in four major steps. All the intermediates and final compounds were purified by recrystallsation as well as column chromatography using Chloroform: Methanol solvent system (9:1). Characterization was performed for the final products from the chalcones following AFO oxidative cyclization using UV visible and a combination of ^1^H and ^13^C NMR. Physical characterisation of all the synthesized compounds are provided in supplementary materials.

### Anti-oxidant studies

Alternatively, the anti-oxidant activity for the test compounds was determined by radical scavenging assays in a cell-free in vitro settings using DPPH. and ABTS.+ . Most of the test compounds were found to show DPPH. and ABTS. + radical scavenging activity as shown in Table 4. The aurones, namely NMA2(2 bromo substituted (methyl series), NEA3 (2 bromo substituted (ethyl series) and NEA4 (4-bromo substituted (ethyl series) showed ABTS. + radical scavenging activity at IC_50_ value of 66.46 ± 2.02, 67.09 ± 3.20 and 71.67 ± 0.34, respectively, and showed DPPH scavenging activity at IC_50_ value of 83.15 ± 6.42, 85.55 ± 6.87and 94.67 ± 4.90, respectively, when compared with that of the standards at the IC_50_ values of 24.13 ± 1.23 and 18.46 ± 1.38 for ascorbic acid, 38.95 ± 0.58 and 31.42 ± 0.09 for the standard Quercetin, respectively. Though, the assay results showed that the anti-oxidant activity of the test compounds were not comparable to that of the standard anti-oxidants used, nevertheless they exhibited moderate antioxidant potentials.

**Table 4:** Antioxidant activity of the synthesized test compounds

Compound code	Radical scavenging activity, IC50 (µM)	
	ABTS^.+^ assay	DPPH^.^ assay
NMA1	130.84 ± 1.74	151.26 ± 3.54
NMA2	66.46 ± 2.02	83.15 ± 6.42
NMA3	89.78 ± 1.48	103.43 ± 7.78
NMA4	137.17 ± 6.23	154.32 ± 12.66
NMA5	87.00 ± 0.18	98.23 ± 1.99
NMA6	200.19 ± 4.43	258.23 ± 8.83
NEA1	120.89 ± 1.08	140.89 ± 13.89
NEA2	179.20 ± 6.00	220.20 ± 2.77
NEA3	67.09 ± 3.20	85.55 ± 6.87
NEA4	71.67 ± 0.34	94.67 ± 4.90
NEA5	> 1000	339 ± 9.65
NEA6	198.18 ± 0.08	222.88 ± 4.87
NPA1	100.97 ± 2.81	132.78 ± 19.09
NPA2	99.32 ± 1.14	118.67 ± 12.87
NPA3	123.07 ± 4.55	158.56 ± 13.89
NPA4	133.56 ± 3.71	162.34 ± 0.06
NPA5	76.12 ± 1.75	89.65 ± 9.65
NISOA1	111.89 ± 5.45	127.87 ± 17.87
NISOA2	234 ± 8.45	381 ± 28.12
NISOA3	187 ± 8.03	284 ± 12.3
NISOA4	127 ± 12.45	159 ± 0.45
NISOA5	109 ± 9.23	129 ± 6.78
NISOA6	146 ± 14.75	183 ± 11.87
Ascorbic acid	24.13 ± 1.23	18.46 ± 1.38
Quercetin	38.95 ± 0.58	31.42 ± 0.09

### Anticancer activity

#### Cytotoxicity using MTT assay

The test compounds were screened for their in vitro cytotoxicity by MTT assay against two cancer cell lines, namely MCF-7, HCT-116 and normal epithelial kidney cells (Vero). Doxorubicin was used as a reference standard.

Many substituted aurones are reported in the literature for their anticancer activities (Anto et al. 1995). In this study, aurones such as **NMA2**, NEA3, NISOA4 and NPA4 showed cytotoxic activity against MCF-7 and HCT 116 when compared to Vero cell lines. However, their cytotoxic activity against breast cancer cell lines (MCF 7)and Colon cancer cell lines(HCT 116) was found to be in the IC50 range of 21.21–43.99 µM when compared with that of the standard doxorubicin at 0.23 ± 0.03–8.32 ± 0.46 µM. Further, the compound **NISOA4** showed selective cytotoxicity towards HCT 116 cell line, while **NMA2** and **NEA3** exhibited better cytotoxic activity on MCF-7 cell lines as shown in Table 5.

**Table 5:** Cytotoxicity of the test compounds on three cell lines

Code	Log D7.4^#^	Inhibition of cell proliferation, IC50 (µM)*		
		MCF-7	HCT 116	Vero
NMA1	2.44	67.82	86.91	186.99
NMA2	3.27	26.76	34.89	153.90
NMA3	2.93	62.67	89.05	188.45
NMA4	2.73	63.77	89.55	> 200
NMA5	2.31	155.43	138.99	> 200
NMA6	2.19	98.54	122.76	> 200
NEA1	3.78	57.88	68.64	ND
NEA2	2.65	49.06	56.73	ND
NEA3	3.61	28.11	32.98	ND
NEA4	3.66	82.65	93.56	187.99
NEA5	2.53	134.76	175.42	176.99
NEA6	3.34	95.43	123.59	191.90
NPA1	4.26	75.01	94.27	165.83
NPA2	3.55	46.87	87.32	172.33
NPA3	4.09	53.87	67.87	ND
NPA4	3.82	42.11	43.99	102.33
NPA5	3.01	68.55	74.32	99.92
NISOA1	4.09	46.78	63.22	172.45
NISOA2	3.93	103.34	124.87	> 200
NISOA3	3.58	70.43	89.54	> 200
NISOA4	2.84	35.65	21.21	> 200
NISOA5	2.97	39.55	55.43	166.94
NISOA6	3.65	43.87	41.77	179.02
Doxorubicin	–	0.68	8.11	164.66

The cytotoxic activity was comparatively more on HCT 116 cell lines when compared to MCF-7 cell lines. Thus, there remains a need to understand the mechanistic studies of most active aurone on HCT 116 cell lines. Based on the existing cytotoxicity data, the compound NISOA4 from aurones series were selected for the detailed mechanistic anticancer studies.

### Comet assay

The Comet Assay, also termed as single cell gel electrophoresis (SCRE) assay, enables the biologists to determine deoxyribonucleic acid (DNA) damage to a single cell from apoptosis (cell death) or the extent of damage caused during cytotoxicity. The DNA of an organism encodes the genetic information that governs both the structure as well as the function of the constituent cells.

**Fig. 11:**
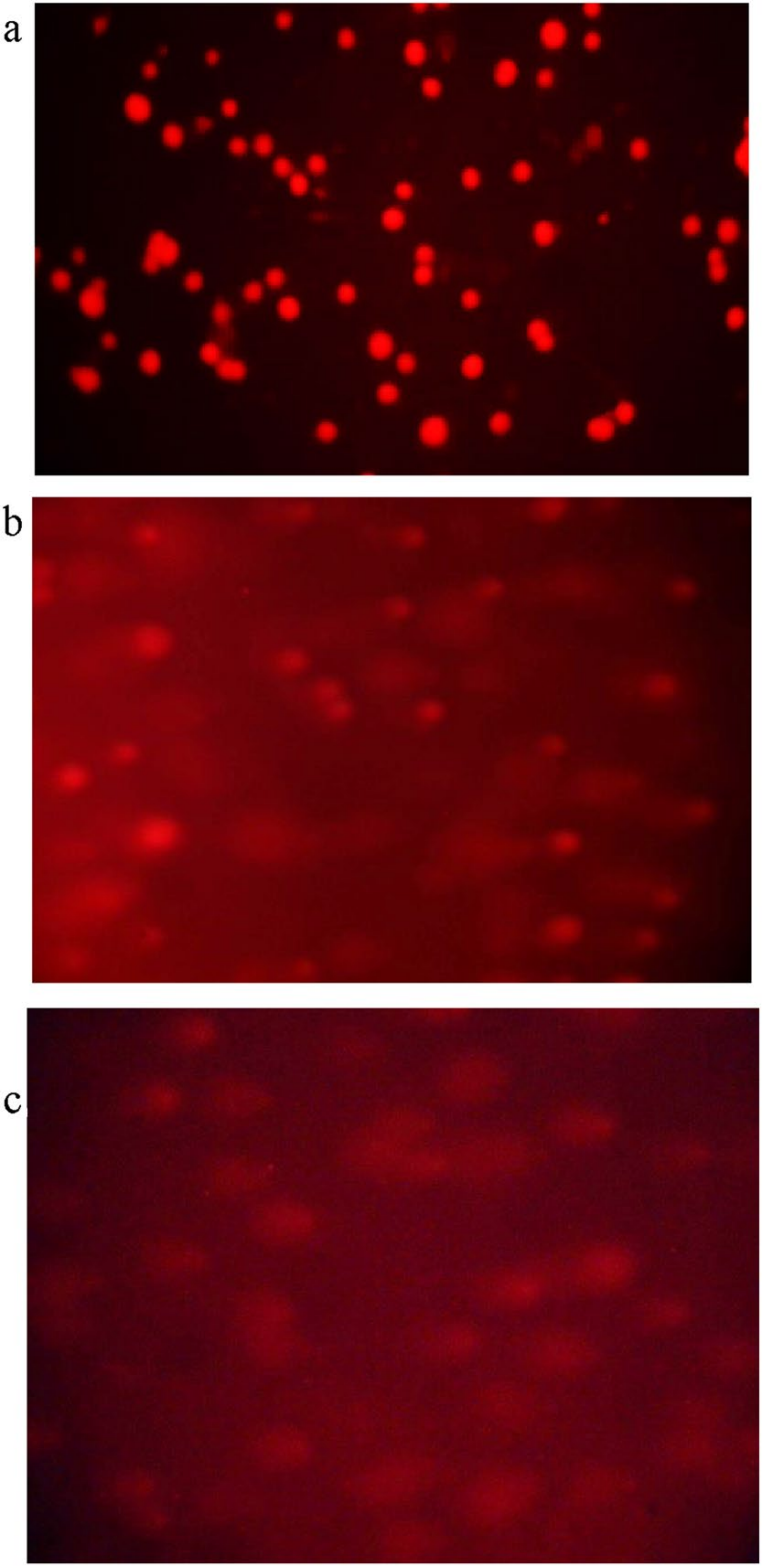
The **a** Comet assay performed in HCT-116 cell lines in the presence of DMSO control. **b** Comet assay performed in HCT-116 cell lines in the presence of sample NISOA4 at a concentration of 12.5 µM. **c** Comet assay performed in HCT-116 cell lines in the presence of sample NISOA4 at a concentration of 25 µM

In the present study, DNA damage was compared within the tested concentration of the samples with untreated control HCT-116 cells. These cells were then treated with NISOA4 that exhibited olive moments of 25.38 and 75.58 at concentration of 12.5 µM and 25 µM as shown in the Fig. 11. Further, sample NISOA4 showed a significant DNA damage in HCT116 cells. Table 6 represents Olive moments observed in cells treated with test samples.

**Table 6:** Olive moments observed in cells treated with test samples

Samples	Olive moments
	(Mean ± SD)
Control	9.206 ± 1.004
NISOA04_12.5	25.384 ± 24.333
NISOA04_25	73.582 ± 97.498

### Apoptotic DNA fragmentation studies

There will be major morphologic changes in the cells and a characteristic form of DNA degradation would be major biochemical hallmark of apoptosis. During apoptosis, various morphologic changes like chromatin condensation and cytoplasmic blebbing take place and are associated with the incidence of nucleosome excision from chromatin. This endonuclease-mediated nucleosome excision is observed as a DNA ladder (multimers of ∼180–200 bp) in agarose gels.

**Fig. 12:**
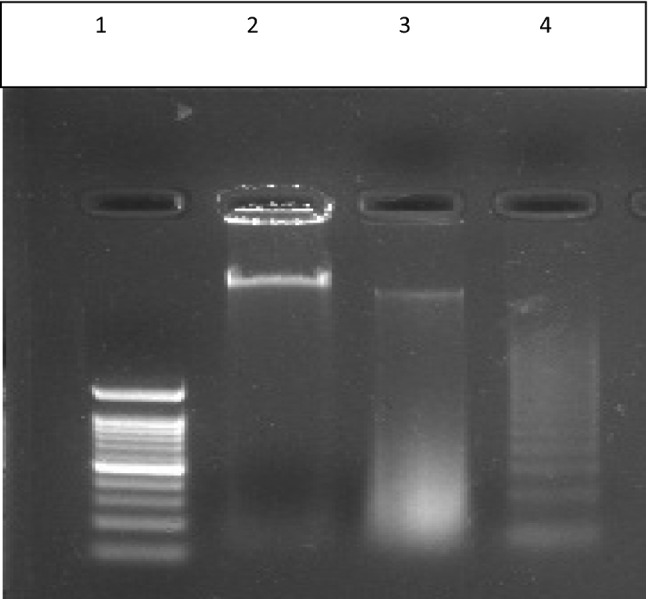
Apoptotic DNA fragmentation in HCT-116 cell lines by the sample NISOA4 lane 1—ladder, lane 2—control, lane 3—12.5 μM, lane 4—25 μM

The HCT 116 cells treated with the sample NISOA4 at concentration of 12.5 μM and 25 μM exhibited DNA Fragmentation. The sample treatment at 12.5 µM showed smearing, whereas the sample treated at 25 µM showed fragmentation in the form of DNA laddering. Overall, these results suggest that the sample NISOA4 exhibited fragmentation of DNA and thus, induced apoptosis as shown in Fig. 12.

### Cell cycle analysis

The eukaryotic cell cycle has been broadly divided into an interphase (G1, S and G2 sub- phases) responsible for the cell division as well as the cellular growth, DNA synthesis, replication and mitotic phase, where the cell division takes place. The progression through different phases of cell cycle is well regulated by a specific cell cycle checkpoints that regulates different stages that are involved in the cell division leading to cell proliferation and cell death (Hartwell and Weinert 1989). The defects in various cell cycle regulatory proteins or key check points could lead to unrestricted cell growth, abnormal development and inhibition of natural cell death mechanisms (apoptosis) and is regarded as the hallmarks in cancer pathogenesis. Anti-cancer agents are being routinely screened for their cytotoxic effect on the cell cycle that depend on their impact on the cell-cycle check points as well as their regulatory proteins.

**Fig. 13:**
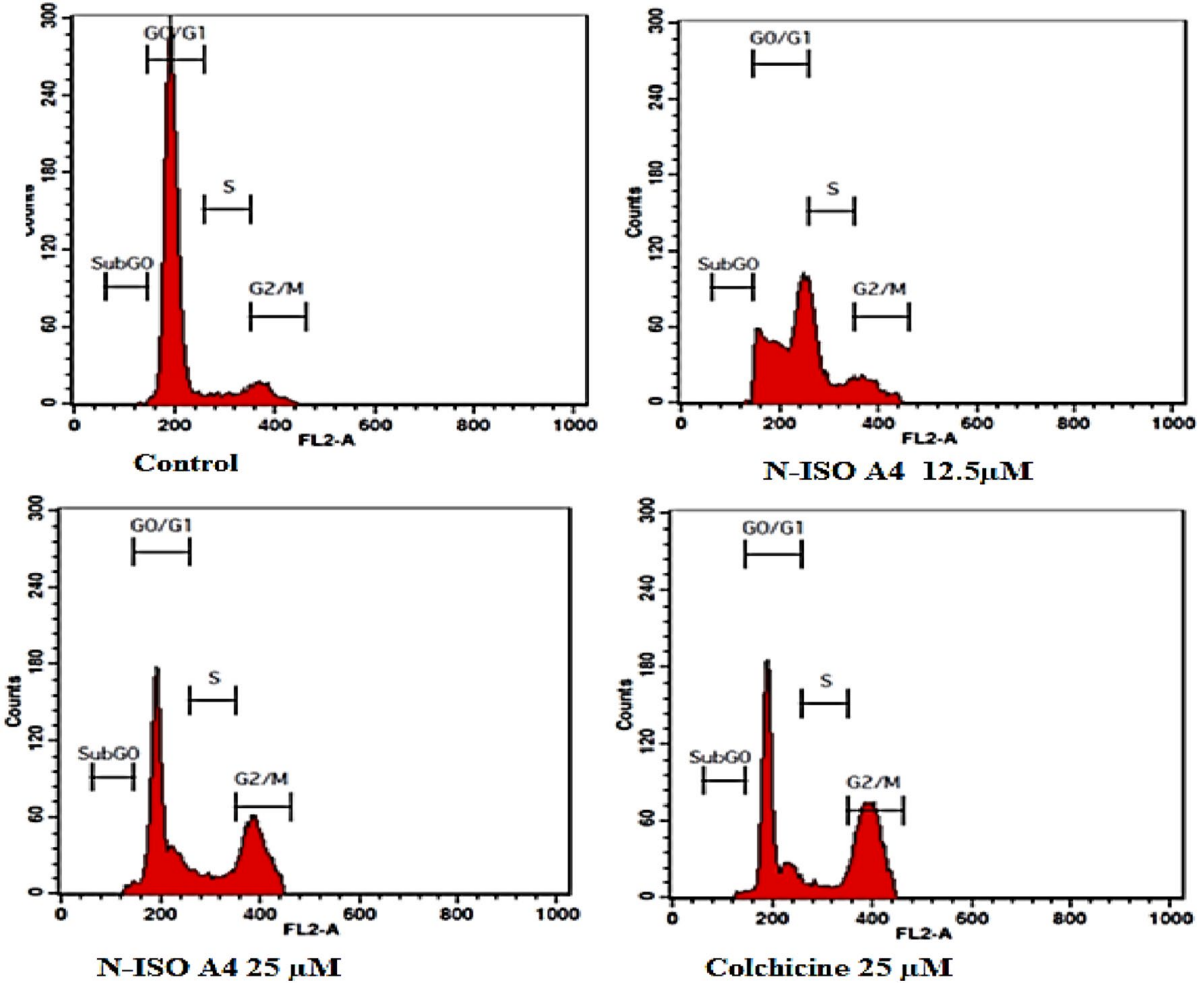
Effect of different concentrations of NISOA4 on cell cycle of HCT 116 cell lines against control and standard colchicine

A 24-h treatment with the test compound NISOA4 at two different concentrations of 12.5 and 25 μg/ml displayed cell cycle arrest at various phases of the cell cycle in comparison to DMSO control treatment. Treatment with low concentration (12.5) showed cell cycle arrest at G_O_/G_1,_S, G_2_/M phase with a percentage cell population at 70.84%, 10.61% 17.30% respectively in comparison to control DMSO (G_O_/G_1_-72.24%, S-10.99% and G_2_/M-16.34%) as shown in Fig. 13.Similarly, treatment with high concentration (25) showed cell cycle arrest G_O_/G_1,_S,G_2_/M phase with a percentage cell population at 57.97%,16.48%,24.98%, respectively, in comparison to control DMSO (G_O_/G_1_-72.24%, S-10.99% and G_2_/M-16.34%).

Colchicine [35] was used as the standard for the comparison of cell cycle arrest with that of test compounds. It showed cell cycle arrest at G_O_/G_1,_ S, and G2/M phase with a percentage cell population at 47.39, 8.61, and 43.78, respectively, at the concentrations of 25 μM as shown in Fig. 13.

#### Apoptosis detection using propidium iodide and annexin V –FITC staining technique

Apoptosis and gene-mediated pathways are one of the important factors that govern the regulatory mechanism associated with the cell survival, proliferation, differentiation and cell death. Mutations, defects or variations in the normal functioning of apoptotic pathways are associated with cancer. Many of the cytotoxic agents are reported to modulate the apoptosis mediated through more than one particular mechanism. As an outcome, it is relevant to study the effect of cytotoxic agents mediated through apoptotic pathway in cancer (Kuntz et al. 1999).

**Fig. 14:**
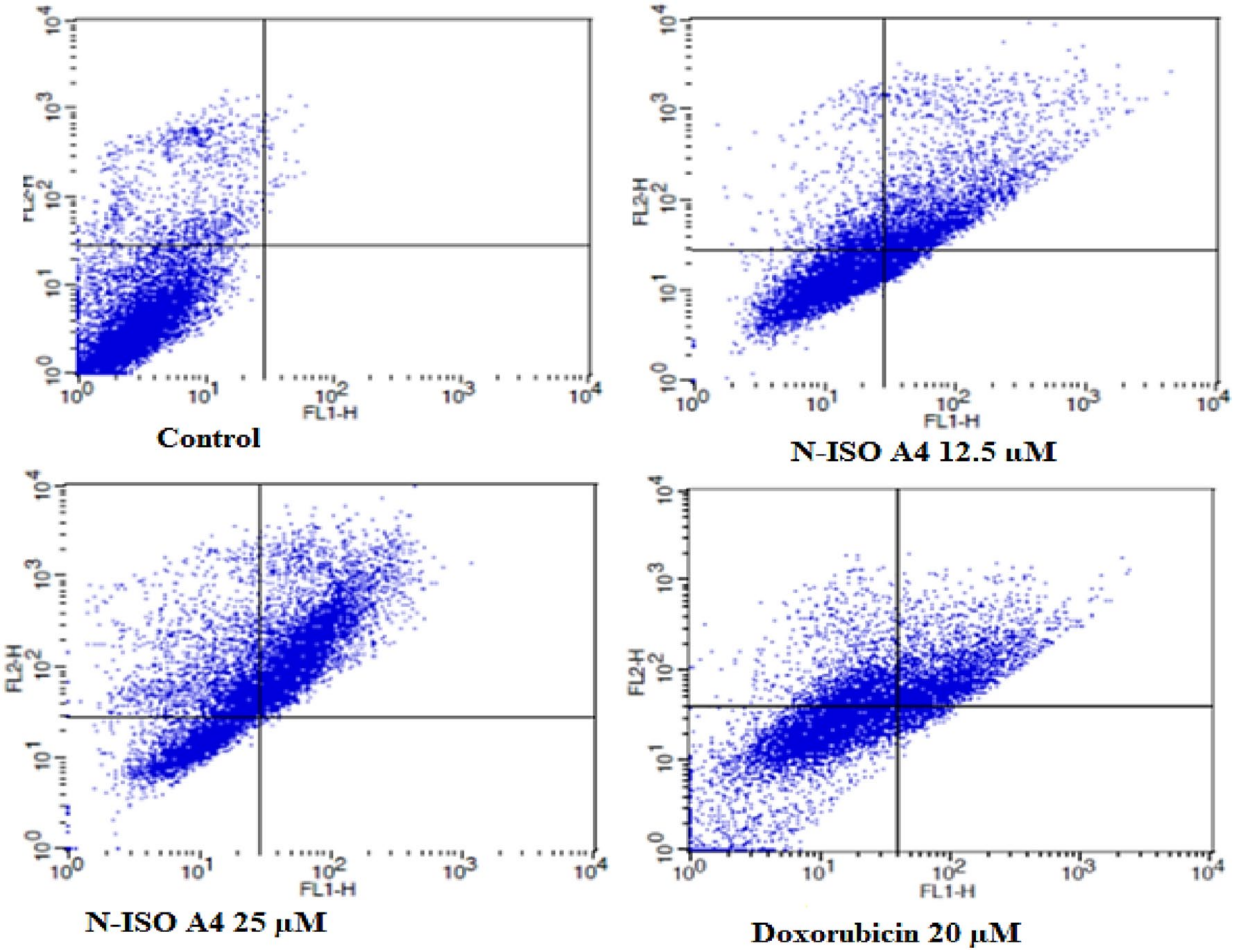
Effect of NISO A4 on the percentage of annexin V-FITC-positive staining in HCT 116 cells. The four quadrants identified as: *LL* viable, *LR* early apoptotic, *UR* late apoptotic, *UL* necrotic

**Fig. 15:**
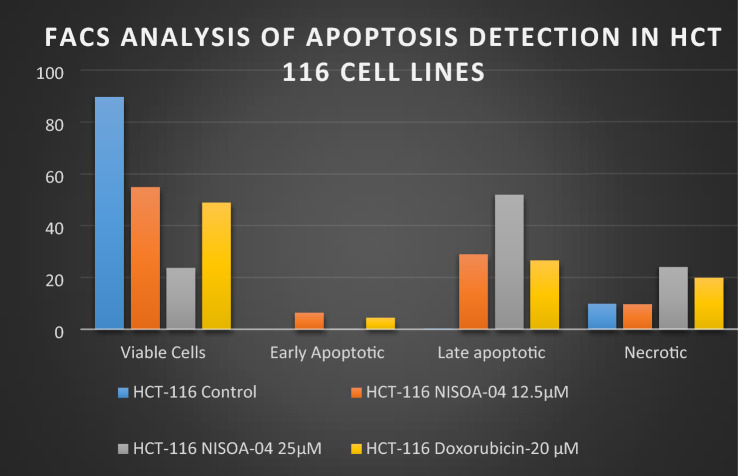
FACS analysis of apoptosis detection in HCT-116 cells treated with test and standard drugs

In this study, aurone, namely **NISOA4** a *p*-methoxy analog was subjected to Propidium iodide/Annexin v –FITC dual staining on HCT-116 cell lines. The extent to which apoptosis was expressed in terms of apoptotic index was calculated as the percentage of apoptotic cells or bodies per all tumor cells. It was found that a 24-h treatment with the test compounds showed characteristic morphological changes in PS asymmetry. That was further analyzed by measuring Annexin V binding to the cell membrane and was detected prior to any morphological alterations that occurred during apoptosis or at the time when the membrane integrity was lost. The aurone **NISOA4** at 12.5 µM, 25 µM showed a significant increase in the apoptotic index at 24.04%, 46.18% and 28.95%, 52.11%, respectively, when compared with that of the media control at 0.37% as shown in Fig. 14.However, the standard doxorubicin showed an apoptotic index of 26.61% at 20 µM as shown in Fig. 14. Among the test compounds evaluated, apoptotic index exhibited by the NISOA4 was found to be the most promising and its apoptotic index was significantly greater than that of the standard doxorubicin (Tyagi et al. 2004) as shown in Table 7. FACS analysis is shown in Fig. 15.

**Table 7:** Flow cytometric analysis of Apoptosis detection of HCT-116 cells

Cell line	Conc	FACS analysis of apoptosis detection in HCT-116 cells			
		Viable cells	Early apoptotic	Late apoptotic	Necrotic
HCT-116	Control	89.73	0	0.37	9.9
	NISOA-04 12.5 µM	55.08	6.36	28.95	9.61
	NISOA-04 25 µM	23.66	0.17	52.11	24.06
	Doxorubicin-20 μM	49.09	4.44	26.61	19.86

#### Semi-quantitative RT-PCR based gene expression studies on CDK2/cyclin A

**Fig. 16:**
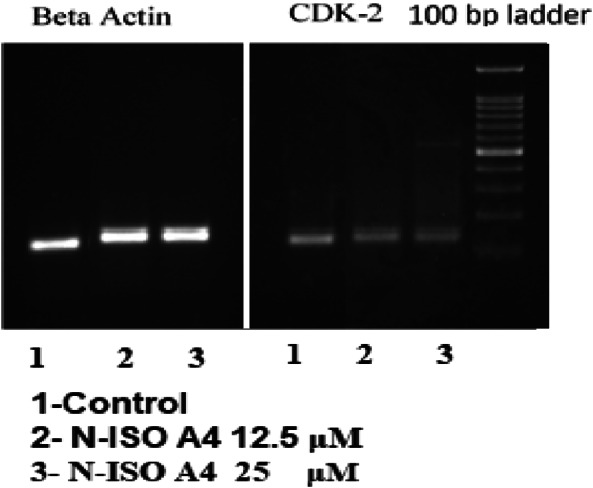
Amplification of beta actin and CDK2 gene in the presence of compound NISOA4 at test concentrations

**Fig. 17:**
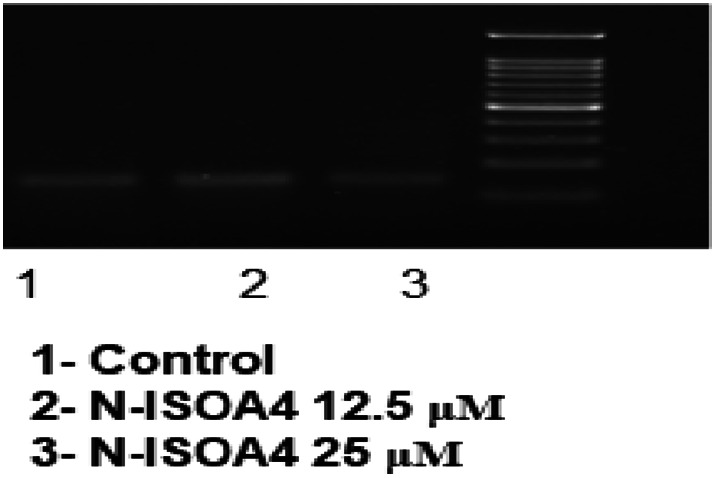
Amplification of Cyclin A gene in the presence NISOA4 at both test concentrations

Gene expression is the process where the information from a gene is used in the synthesis of a functional gene product. The investigation monitors the response of a gene for the treatment with a compound or a drug of interest, under a defined set of conditions (Haber et al. 1999).Gene Expression for CDK2 and CyclinA was carried out in HCT 116 cell lines for the test compound NISOA4 at the concentration of 12.5 μM and 25 ,μM respectively. There exists a clear dose-dependent decrease in the expression of CDK2 at 12.5 μM and 25 μM concentration. However, CyclinA expression profile showed very little or no reduction in the 12.5 μM concentration, whereas there was a clear decrease in the 25-μM concentration treatment. Amplification of beta actin and CDK2 gene is shown in Fig. 16, amplification (Shen et al. 1986) of cyclin A gene is shown in the Fig. 17, relative expression of CDK2 is shown in Table 8 and relative expression of Cyclin A is shown in Table 9.

**Table 8:** Relative expression of CDK2 in HCT 116 cell lines

Samples	Band intensity of PCR amplicon of genes		Normalized	Relative gene expression
	β-Actin	CDK2		
Control	22,612.953	24,012.146	1.06	1.00
NISOA4-12.5	25,501.761	16,091.61	0.63	0.59
NISOA4- 25	25,292.075	13,190.418	0.52	0.49

**Table 9:** Relative expression of Cyclin A in HCT 116 cell lines

Samples	Band intensity Of PCR Amplicon Of genes		Normalized	Relative gene expression
	β-Actin	Cyclin A		
Control	22,612.95	14,414.016	0.64	1.00
NISOA4 12.5	25,501.76	16,245.903	0.64	1.00
NISO A4- 25	25,292.08	10,798.368	0.43	0.67

#### Inhibitory activity of CDK2/cyclin A enzyme target using Z´-Lyte™ kinase assay kit—ser/thr 12 peptide

CDKs have emerged out as one of the important cancer drug targets in recent years owing to their specificity on different phases of the cell cycle (Noble et al. 2004). In particular, the CDK2/Cyclin A acts predominantly on G2/M phase of the cell cycle that has attracted interest amongst many biologists. Several distinct CDK2 inhibitors are currently undergoing clinical trials, and some of the classes of these drugs have been already approved. (Schiele et al. 2015).

It is noteworthy to mention the importance of flavopiridol in this context. It belongs to the class of flavonoids and is currently existing as an investigational drug. This drug has been given an approval for the treatment of CLL under fast track approval amongst the European Union. Its inefficiency as a single drug in many clinical studies has limited its usage in clinical therapy.

In the present study, it was thought to synthesize novel analogues of flavopiridol to achieve increased potency and lowered toxicity.

The CDK2/Cyclin A assay was carried out as per the manufacturer’s instruction using suitable controls and standard.

**Fig. 18:**
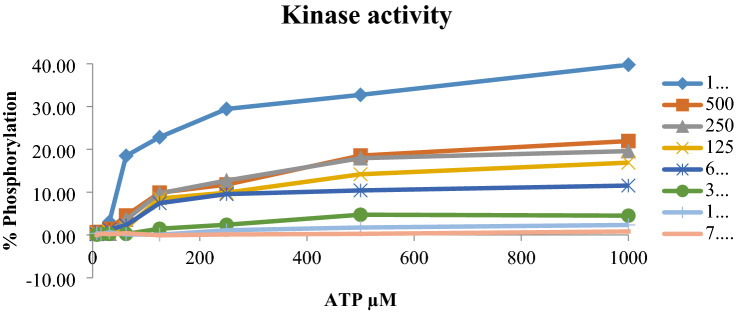
Optimization of kinase and ATP at varying concentrations

Before carrying out any kinase assay, it is required to standardize kinase as well as ATP to set an optimum concentration so as to fix particular percentage phosphorylation. In the present study, it was found that a maximal activity of kinase and ATP was found to be at 1000 ng/ml kinase concentration and 1000 µM ATP with phosphorylation of 39.77% as shown in Table 10 and Fig. 18.

**Table 10:** Optimization of kinase and ATP at varying concentrations

% Phosphorylation	ATP µM							
Kinase ng/ml	1000	500	250	125	62.5	31.25	15.63	7.81
1000	39.77	32.74	29.44	22.86	18.49	3.05	0.70	0.47
500	21.94	18.54	11.77	9.88	4.54	1.42	0.67	0.65
250	19.59	17.94	12.71	9.72	3.59	0.29	0.42	0.28
125	16.90	14.18	9.88	8.53	1.63	0.84	0.49	0.50
62.50	11.54	10.41	9.55	7.43	2.27	1.27	0.60	0.21
31.25	4.52	4.73	2.39	1.46	0.24	0.24	0.24	0.07
15.63	2.34	1.74	1.11	0.06	0.13	0.62	0.24	0.40
7.81	0.82	0.26	0.12	− 0.08	0.41	0.20	0.31	− 0.03
Controls	0.00	100.00

The inhibitory assay was carried out using the stock of 10 mM in DMSO. Samples were further serially diluted to obtain concentrations of 100 μM. The concentrations were chosen based on the IC50 values obtained in the MTT assay. A total of 23 analogues were selected for the assay based on their antiproliferative activity. Further, selected compounds were screened and tested against the target CDK2/Cyclin A, and their % phosphorylation and % inhibition were recorded. Roscovitine was used as standard and 0%, 100% phosphorylation controls were used. In the present study, compounds from isopropyl series showed appreciable inhibition against CDK2/Cyclin A (38–43%), whereas methyl and ethyl series also showed inhibition at the percentage of 25–35%. The propyl series showed lower percentage inhibition against the target CDK2/Cyclin A (12–15%). The percentage inhibition values and free energy of binding values are compared in Table 11.

**Table 11:** CDK2/cyclin A inhibitory activity of test compounds at a concentration of 100 micro molar along with their binding free energy

Compound	% inhibition on CDK2/cyclin A	MMGBSA dG bind (Kcal/mol)
NMA1	27.654	− 41.65
NMA2	35.350	− 51.88
NMA3	24.987	− 35.61
NMA4	29.543	− 37.88
NMA5	25.098	− 36.98
NMA6	26.113	− 37.99
NEA1	32.676	− 43.63
NEA2	28.008	− 46.81
NEA3	35.559	− 52.20
NEA4	27.236	− 42.75
NEA5	26.007	− 43.50
NEA6	25.879	− 42.52
NPA1	16.089	− 36.90
NPA2	13.664	− 34.43
NPA3	17.045	− 36.71
NPA4	14.753	− 35.51
NPA5	12.547	− 32.56
NISOA1	38.987	− 58.73
NISOA2	39.811	− 51.08
NISOA3	43.654	− 50.32
NISOA4	42.787	− 58.45
NISOA5	39.054	− 57.54
NISOA6	36.546	− 55.30
Roscovitine	80.55	ND

## Conclusion

The present research finding has led to the design and synthesis of novel aurone analogues and establishing their chemistry. Though, we extensively studied their anti-oxidant and anti-cancer potential there is a huge scope in exploiting other therapeutic potentials The test compound NISOA4, a isopropyl piperidinyl derivative, emerged out as a potential candidate against colon cancer in all the cell-based as well as FRET assays. This study created an insight for understanding the mechanistic studies on homologues series of N-alkyl (methyl, ethyl, propyl, isopropyl) tetrahydropyridinyl aurones against anticancer target CDK2/Cyclin A in colon cancer. However, in vivo studies are yet to be demonstrated to assess their anticancer potential in animal models. Further, future resides in developing aurone analogs to be evaluated on the basis of antibody based enzyme assays to reconfirm their potential to inhibit CDK2/Cyclin A and perform animal studies to develop them as a clinical drug candidates and also their potential for DNA intercalation has to be exploited.

## Supplementary Information

Below is the link to the electronic supplementary material.

Supplementary file1 (DOCX 233 kb)

## Abbreviations

CDK: Cyclin dependent kinase
MTT assay: 3-(4,5-Dimethylthiazol-2-yl)-2,5-diphenyl tetrazolium bromide assay
IC_50_: Half-maximal inhibitory concentration
FITC: Ferrous isothiocyanate
DNA: Deoxy ribonucleic acid
RTPCR: Reverse transcriptase polymerase chain reaction

## Declarations

### Research involves human or animal participant

Not applicable to the current research as we have not used any human or animal subject.

## References

[CR1] Ahn YM, Vogeti L, Liu CJ, Santhapuram HK, White JM, Vasandani V, Mitscher LA, Lushington GH, Hanson PR, Powell DR, Himes RH (2007). Design, synthesis, and antiproliferative and CDK2-cyclin a inhibitory activity of novel flavopiridol analogues. Bioorg Med Chem.

[CR2] Anto RJ, Sukumaran K, Kuttan G, Rao MN, Subbaraju V, Kuttan R (1995). Anticancer and antioxidant activity of synthetic chalcones and related compounds. Cancer Lett.

[CR3] Bhardwaj VK, Singh R, Sharma J, Das P, Purohit R (2020). Structural based study to identify new potential inhibitors for dual specificity tyrosine-phosphorylation-regulated kinase. Comput Methods Programs Biomed.

[CR4] Bookout AL, Mangelsdorf DJ (2003). Quantitative real-time PCR protocol for analysis of nuclear receptor signaling pathways. Nucl Recept Signal.

[CR5] Chomczynski P, Sacchi N (1987). Single-step method of RNA isolation by acid guanidinium thiocyanate-phenol-chloroform extraction. Anal Biochem.

[CR6] Cohen P (1999). The development and therapeutic potential of protein kinase inhibitors. Curr Opin Chem Biol.

[CR7] Collins AR (2005). Assays for oxidative stress and antioxidant status: applications to research into the biological effectiveness of polyphenols. Am J Clin Nutr.

[CR8] Engh RA, Bossemeyer D (2001). The protein kinase activity modulation sites: mechanisms for cellular regulation-targets for therapeutic intervention. Adv Enzym Regul.

[CR9] Friesner RA, Murphy RB, Repasky MP, Frye LL, Greenwood JR, Halgren TA, Sanschagrin PC, Mainz DT (2006). Extra precision glide: docking and scoring incorporating a model of hydrophobic enclosure for protein—ligand complexes. J Med Chem.

[CR10] Haber M, Bordow SB, Gilbert J, Madafiglio J, Kavallaris M, Marshall GM, Mechetner EB, Fruehauf JP, Tee L, Cohn SL, Salwen H (1999). Altered expression of the MYCN oncogene modulates MRP gene expression and response to cytotoxic drugs in neuroblastoma cells. Oncogene.

[CR11] Hartwell LH, Weinert TA (1989). Checkpoints: controls that ensure the order of cell cycle events. Science.

[CR12] Jayashree B, Nigam S, Pai A, Patel H, Reddy N, Kumar N, Rao CM (2015). Targets in anticancer research—a review. Indian J Exp Biol.

[CR13] Jia Y, Quinn CM, Kwak S, Talanian RV (2008). Current in vitro kinase assay technologies: the quest for a universal format. Curr Drug Discov Technol.

[CR14] Kasibhatla S, Amarante-Mendes GP, Finucane D, Brunner T, Bossy-Wetzel E, Green DR (2006). Acridine orange/ethidium bromide (AO/EB) staining to detect apoptosis. Cold Spring Harb Protoc.

[CR15] Kaur G, Stetler-Stevenson M, Sebers S, Worland P, Sedlacek H, Myers C, Czech J, Naik R, Sausville E (1992). Growth inhibition with reversible cell cycle arrest of carcinoma cells by flavone L86–8275. J Natl Cancer Inst.

[CR16] Kim KS, Sack JS, Tokarski JS, Qian L, Chao ST, Leith L, Kelly YF, Misra RN, Hunt JT, Kimball SD, Humphreys WG (2000). Thio-and oxoflavopiridols, cyclin-dependent kinase 1-selective inhibitors: synthesis and biological effects. J Med Chem.

[CR17] Końca K, Lankoff A, Banasik A, Lisowska H, Kuszewski T, Góźdź S, Koza Z, Wojcik A (2003). A cross-platform public domain PC image-analysis program for the comet assay. Mutat Res Genet Toxicol Environ Mutagen.

[CR18] Kuntz S, Wenzel U, Daniel H (1999). Comparative analysis of the effects of flavonoids on proliferation, cytotoxicity, and apoptosis in human colon cancer cell lines. Eur J Nutr.

[CR19] Lebakken CS, Kang HC, Vogel KW (2007). A fluorescence lifetime–based binding assay to characterize kinase inhibitors. J Biomol Screen.

[CR20] Lee MG, Nurse P (1987). Complementation used to clone a human homologue of the fission yeast cell cycle control gene cdc2. Nature.

[CR21] Meijer L, Borgne A, Mulner O, Chong JP, Blow JJ, Inagaki N, Inagaki M, Delcros JG, Moulinoux JP (1997). Biochemical and cellular effects of roscovitine, a potent and selective inhibitor of the cyclin-dependent kinases cdc2, cdk2 and cdk5. Eur J Biochem.

[CR22] Morgan DO (1997). Cyclin-dependent kinases: engines, clocks, and microprocessors. Annu Rev Cell Dev Biol.

[CR23] Murakami Y (1996). Reverse transcriptase-polymerase chain reaction. Tanpakushitsu kakusan koso. Protein, nucleic acid, enzyme. Foodborne Pathog Dis.

[CR24] Nigam S, Jayashree BS (2017). Limitation of Algar–Flynn–Oyamada reaction using methoxy substituted chalcones as reactants and evaluation of the newly transformed aurones for their biological activities. Res Chem Intermed.

[CR25] Noble ME, Endicott JA, Johnson LN (2004). Protein kinase inhibitors: insights into drug design from structure. Science.

[CR26] Nurse P, Bissett Y (1981). Gene required in G1 for commitment to cell cycle and in G 2 for control of mitosis in fission yeast. Nature.

[CR27] Schiele F, Ayaz P, Fernández-Montalván A (2015). A universal homogeneous assay for high-throughput determination of binding kinetics. Anal Biochem.

[CR28] Semenov I, Akyuz C, Roginskaya V, Chauhan D, Corey SJ (2002). Growth inhibition and apoptosis of myeloma cells by the CDK inhibitor flavopiridol. Leukemia Res.

[CR29] Serizawa H, Mäkelä TP, Conaway JW, Conaway RC, Weinberg RA, Young RA (1995). Association of Cdk-activating kinase subunits with transcription factor TFIIH. Nature.

[CR30] Shen DW, Fojo A, Chin JE, Roninson IB, Richert N, Pastan I, Gottesman MM (1986). Human multidrug-resistant cell lines: increased mdr1 expression can precede gene amplification. Science.

[CR31] Singh R, Bhardwaj V, Das P, Purohit R (2020). Natural analogues inhibiting selective cyclin-dependent kinase protein isoforms: a computational perspective. J Biomol Struct Dyn.

[CR32] Singh R, Bhardwaj VK, Sharma J, Das P, Purohit R (2021). Discovery and in silico evaluation of aminoarylbenzosuberene molecules as novel checkpoint kinase 1 inhibitor determinants. Genomics.

[CR33] Singh R, Bhardwaj VK, Sharma J, Das P, Purohit R (2021). Identification of selective cyclin-dependent kinase 2 inhibitor from the library of pyrrolone-fused benzosuberene compounds: an in silico exploration. J Biomol Struct Dyn.

[CR34] Singh R, Bhardwaj VK, Purohit R (2022). Computational targeting of allosteric site of MEK1 by quinoline-based molecules. Cell Biochem Function.

[CR35] Tice RR, Agurell E, Anderson D, Burlinson B, Hartmann A, Kobayashi H, Miyamae Y, Rojas E, Ryu JC, Sasaki YF (2000). Single cell gel/comet assay: guidelines for in vitro and in vivo genetic toxicology testing. Environ Mol Mutagen.

[CR36] Tyagi AK, Agarwal C, Chan DC, Agarwal R (2004). Synergistic anti-cancer effects of silibinin with conventional cytotoxic agents doxorubicin, cisplatin and carboplatin against human breast carcinoma MCF-7 and MDA-MB468 cells. Oncol Rep.

[CR37] Tyers M (2004). Cell cycle goes global. Curr Opin Cell Biol.

[CR38] Van Meerloo J, Kaspers GJ, Cloos J (2011). Cell sensitivity assays: the MTT assay. Cancer cell culture.

[CR39] Vermes I, Haanen C, Steffens-Nakken H, Reutellingsperger C (1995). A novel assay for apoptosis flow cytometric detection of phosphatidylserine expression on early apoptotic cells using fluorescein labelled annexin V. J Immunol Methods.

[CR40] Zheleva-Dimitrova D, Nedialkov P, Kitanov G (2010). Radical scavenging and antioxidant activities of methanolic extracts from Hypericum species growing in Bulgaria. Pharmacogn Mag.

[CR41] Liu X, Go ML (2006) Antiproliferative properties of piperidinyl chalcones. Bioorg. Med. Chem 14(1):153–163. 10.1016/j.bmc.2005.08.006 10.1016/j.bmc.2005.08.006 16185876

[CR42] Narsinghani T, Sharma M C, Bhargav S (2013) Synthesis, docking studies and antioxidant activity of some chalcone and aurone derivatives. Med Chem Res, 22(9):4059–4068.

