# Supplementary material for: Novel benzylidene benzofuranone analogues as potential anticancer agents: design, synthesis and in vitro evaluation based on CDK2 inhibition assays
Source: 3 Biotech. 2022 Sep 2;12(10):256. doi: 10.1007/s13205-022-03312-1 (PMC9440176; doi:10.1007/s13205-022-03312-1)
Supplement: Supplementary file 1 — Supplementary file1 (DOCX 233 kb) [file 13205_2022_3312_MOESM1_ESM.docx]

**Supplementary information to**

**Original Article**

**NOVEL BENZYLIDENE BENZOFURANONE ANALOGUES AS POTENTIAL ANTICANCER AGENTS: DESIGN, SYNTHESIS AND IN VITRO EVALUATION BASED ON CDK2 INHIBITION ASSAYS.**

**Table of Contents**

**Spectral characterization data for the compounds NMA1- NISOA6**

**Structure of synthesized compounds**

**Spectral details of the synthesized test compounds**

(2Z)-2-benzylidene-7-(1,2,3,6-tetrahydro-1-methylpyridin-4-yl)-4,6-dimethoxybenzofuran3(2H)-one **(NMA1)** C_23_H_23_NO_4_ **,** yellow solid, yield 72%,UV: λmax (MeOH) 378 nm (ε3.7770x10^3^cm^-1^M^-1^ ) ^1^H NMR(400 MHZ) : δ 2.25-2.40 (5H, 2.29 (s), 2.33 (ddd, *J* = 14.9, 10.1, 3.8 Hz)), 2.72 (2H, ddd, *J* = 13.8, 3.8, 2.0 Hz), 3.10 (2H, dd, *J* = 13.9, 3.6 Hz), 3.82-3.83 (6H, 3.82 (s), 3.82 (s)), 6.03 (1H, dd, *J* = 5.0, 3.6 Hz), 6.23 (1H, s), 7.26 (1H, tt, *J* = 7.5, 1.6 Hz), 7.36-7.46 (5H, 7.39 (s), 7.42 (dddd, *J* = 8.1, 2.1, 1.6, 0.5 Hz), 7.41 (dddd, *J* = 8.1, 7.5,1.9,0.5Hz).^13^CNMR(400MHz,DMSOd6):179.03,162.81,158.97,157.28,143.37,135.60,132.22,131.11,129.42,128.72,120.77,112.05,109.80105.77,93.52,56.52,56.41,56.23,53.29,45.44,28.37, MS (APCI): *m/z* 378.55 [M+1]^+^ (100%).

(2Z)-2-(2-bromobenzylidene)-7-(1,2,3,6-tetrahydro-1-methylpyridin-4-yl)-4,6 dimethoxybenzofuran- 3(2H)-one **(NMA2)** C_23_H_22_BrNO_4_, pale brown crystals, yield 69% , UV: λmax (MeOH) 386.56 nm (ε3.5643x10^3^cm^-1^M^-1^). ^1^H NMR: δ 2.25-2.41 (5H, 2.29 (s), 2.33 (ddd, *J* = 14.9, 10.1, 3.8 Hz)), 2.72 (2H, ddd, *J* = 13.8, 3.8, 2.0 Hz), 3.10 (2H, dd, *J* = 13.9, 3.6 Hz), 3.82-3.83 (6H, 3.82 (s), 3.82 (s)), 6.03 (1H, dd, *J* = 5.0, 3.6 Hz), 6.23 (1H, s), 7.22 (1H, ddd,*J*= 8.1,7.3,1.7Hz),7.41,7.59(4H,7.45(ddd,*J*=8.2,1.7,0.5Hz),7.48(s),7.50(ddd,*J*=8.2,7.3,1.5Hz),7.56(ddd, *J* =8.1,1.5,0.5Hz),^13^CNMR(400MHz,DMSOd6):178.46,162.83,158.97,157.28,144.02,135.60,134.16,132.23,132.15,129.88,126.64,126.11,120.77,109.80,107.18,105.79,93.52,56.52,56.41,56.23,53.29,45.44,28.37, MS (APCI): *m/z* 457.48 [M+1]^+^ (100%).

2Z)-2-(2-methylbenzylidene)-7-(1,2,3,6-tetrahydro-1-methylpyridin-4-yl)-4,6 dimethoxybenzofuran-3(2H)-one**(NMA3)** C24H25NO4, light yellow crystals, yield 67%, UV: λmax (MeOH) 401 nm (ε4.9344x10^4^cm^-1^M^-1^). ^1^H NMR: δ 2.25-2.40 (8H, 2.26 (s), 2.33 (ddd, *J* = 14.9, 10.1, 3.8 Hz), 2.29 (s)), 2.72 (2H, ddd, *J* = 13.8, 3.8, 2.0 Hz), 3.10 (2H, dd, *J* = 13.9, 3.6 Hz), 3.82-3.83 (6H, 3.82 (s), 3.82 (s)), 6.03 (1H, dd, *J* = 5.0, 3.6 Hz), 6.23 (1H, s), 7.04-7.24 (3H, 7.09 (ddd, *J* = 7.9, 7.7, 2.6 Hz), 7.13 (ddd, *J* = 7.9, 7.7, 1.4 Hz), 7.21 (ddd, *J* = 7.9, 2.6, 0.4 Hz)), 7.46-7.52(2H,7.49(ddd, *J* =7.9,1.4,0.4Hz),7.49(s).^13^CNMR(400MHz,DMSOd6):178.40,162.83,158.97,157.28,142.47,138.86,135.60,133.45,131.76,130.06,129.80,125.53,120.77,109.80,107.09,105.79,93.52,56.52,56.42,56.22,53.29,45.44,28.37,19.41, MS (APCI): *m/z* 392.5.51 [M+1]^+^ (100%).

(2Z)-2-(4-(dimethylamino)benzylidene)-7-(1,2,3,6-tetrahydro-1-methylpyridin-4-yl)-4,6-dimethoxybenzofuran-3(2H)-one **(NMA4)** C_25_H_28_N_2_O_4_**,** Light orange powder**,** yield 78 %,UV: λmax (MeOH) 412.76 nm (ε3.4444x10^3^cm^-1^M^-1^). ^1^H NMR: δ 2.26-2.42 (5H, 2.29 (s), 2.34 (ddd, *J* = 14.9, 10.1, 3.8 Hz)), 2.72 (2H, ddd, *J* = 13.8, 3.8, 2.0 Hz), 2.87 (6H, s), 3.10 (2H, dd, *J* = 13.9, 3.6 Hz), 3.81-3.82 (6H, 3.82 (s), 3.82 (s)), 6.03 (1H, dd, *J* = 5.0, 3.6 Hz), 6.22 (1H, s), 6.72 (2H,ddd,*J*=8.1,1.1,0.5Hz),7.27(1H,s),7.49(2H,ddd,*J*=8.1,1.5,0.5Hz).^13^CNMR(400MHz,DMSOd6):179.03,162.81,158.97,157.28,151.70,143.40,135.60,131.64,124.58,120.77,112.21,112.03,109.80,105.77,93.52,56.52,56.41,56.23,53.29,45.44,40.27,28.37, MS (APCI): *m/z* 421.62 [M+1]^+^ (100%).

(2Z)-2-(4-methoxybenzylidene)-7-(1,2,3,6-tetrahydro-1-methylpyridin-4-yl)-4,6-dimethoxybenzofuran-3(2H)-one **(NMA5)** C_24_H_25_NO_5_, yellow crystals, yield 55%, UV: λmax (MeOH) 399.98 nm (ε3.9876x10^3^cm^-1^M^-1^). ^1^H NMR: δ 2.25-2.41 (5H, 2.29 (s), 2.33 (ddd, *J* = 14.9, 10.1, 3.8 Hz)), 2.72 (2H, ddd, *J* = 13.8, 3.8, 2.0 Hz), 3.10 (2H, dd, *J* = 13.9, 3.6 Hz), 3.79 (3H, s), 3.81-3.83 (6H, 3.82 (s), 3.82 (s)), 6.03 (1H, dd, *J* = 5.0, 3.6 Hz), 6.23 (1H, s), 7.22 (2H, ddd, *J* =8.8,1.2,0.4Hz),7.32(1H,s),7.79(2H,ddd,*J*=8.8,1.7,0.4Hz),^13^CNMR(400MHz,DMSOd6):179.03,162.81,160.43,158.97,157.28,143.40,135.60,132.39,125.93,120.77,113.69,111.95,109.80,105.77,93.52,56.52,56.41,56.23,55.32,53.29,45.44,28.37, MS (APCI): *m/z* 408.56 [M+1]^+^ (100%).

(2Z)-2-(3,4-dimethoxybenzylidene)-7-(1,2,3,6-tetrahydro-1-methylpyridin-4-yl)-4,6-dimethoxybenzofuran-3(2H)-one **(NMA6)** C_25_H_27_NO_6_,pale yellow crystals, yield 60%, UV: λmax (MeOH) 422 nm (ε3.5897x10^3^cm^-1^M^-1^ ). ^1^H NMR: δ 2.25-2.41 (5H, 2.29 (s), 2.33 (ddd, *J* = 14.9, 10.1, 3.8 Hz)), 2.72 (2H, ddd, *J* = 13.8, 3.8, 2.0 Hz), 3.10 (2H, dd, *J* = 13.9, 3.6 Hz), 3.81-3.83 (9H, 3.82 (s), 3.81 (s), 3.82 (s)), 3.86 (3H, s), 6.03 (1H, dd, *J* = 5.0, 3.6 Hz), 6.23 (1H, s), 6.71 (1H, dd, *J* = 8.4, 0.4 Hz), 7.29 (1H, s), 7.33 (1H, dd, *J* = 1.9, 0.4 Hz), 7.65 (1H, dd, *J* = 8.4, 1.9 Hz),^13^CNMR(400MHz,DMSOd6):162.81,158.97,157.28,150.24,149.65,143.68,135.60,127.31,127.18,120.77,114.10,112.11,111.45,109.81,105.77,93.52,56.52,56.41,56.23,55.90,55.88,53.29,45.44,28.37, MS (APCI): *m/z* 438.52 [M+1]^+^ (100%).

(2Z)-7-(1-ethyl-1,2,3,6-tetrahydropyridin-4-yl)-4,6-dimethoxy-2-((naphthalen-3-yl)methylene)benzofuran-3(2H)-one **(NEA1)** C_28_H_27_NO_4_,pale yellow crystals, yield 74%, UV: λmax (MeOH) 432 nm (ε3.6654x10^3^cm^-1^M^-1^). ^1^H NMR: δ 0.98 (3H, t, *J* = 4.8 Hz), 2.34 (2H, ddd, *J* = 14.9, 10.1, 3.8 Hz), 2.65 (2H, q, *J* = 4.8 Hz), 2.74 (2H, ddd, *J* = 11.6, 3.8, 2.0 Hz), 3.13 (2H, dd, *J* = 13.9, 3.7 Hz), 3.81-3.82 (6H, 3.82 (s), 3.82 (s)), 6.04 (1H, dd, *J* = 5.1, 3.7 Hz), 6.23 (1H, s), 7.50 (1H, dddd, *J* = 8.0, 6.9, 1.9, 0.4 Hz), 7.59 (1H, dddt, *J* = 8.4, 2.5, 0.5, 0.4 Hz), 7.60-7.70 (2H, 7.64 (dddd, *J* = 8.0, 6.9, 2.6, 0.4 Hz), 7.67 (ddd, *J* = 8.4, 2.0, 0.4 Hz)), 7.70-7.84 (4H, 7.75 (s), 7.73 (dddt, *J* = 8.0, 2.1, 1.9, 0.5 Hz), 7.78 (dtq, *J* = 8.0, 2.5, 0.4 Hz), 7.83 (ddq, *J* = 2.1, 2.0,0.4Hz),^13^CNMR(400MHz,DMSOd6):178.53,162.83,158.97,157.28,143.17,135.72,134.29,133.85,131.24,130.45,127.94,127.71,126.52,126.49,125.70,121.39,109.80,105.79,105.73,93.52,56.41,56.23,52.53,50.82,49.81,28.49,11.73, MS (APCI): *m/z* 442.52 [M+1]^+^ (100%).

(2Z)-2-(2-methoxybenzylidene)-7-(1-ethyl-1,2,3,6-tetrahydropyridin-4-yl)-4,6-dimethoxybenzofuran-3(2H)-one **(NEA2)** C_25_H_27_NO_5_**,**yellow crystals, yield 62%, UV: λmax (MeOH) 415 nm (ε4.6666x10^3^cm^-1^M^-1^). ^1^H NMR: δ 0.98 (3H, t, *J* = 4.8 Hz), 2.34 (2H, ddd, *J* = 14.9, 10.1, 3.8 Hz), 2.65 (2H, q, *J* = 4.8 Hz), 2.74 (2H, ddd, *J* = 11.6, 3.8, 2.0 Hz), 3.13 (2H, dd, *J* = 13.9, 3.6 Hz), 3.81-3.83 (6H, 3.82 (s), 3.82 (s)), 3.85 (3H, s), 6.03 (1H, dd, *J* = 5.1, 3.6 Hz), 6.23 (1H, s), 7.15-7.25 (2H, 7.20 (ddd, *J* = 8.0, 7.4, 1.2 Hz), 7.20 (ddd, *J* = 8.0, 1.2, 0.5 Hz)), 7.33 (1H, ddd, *J* =8.0,7.4,1.3Hz),7.42(1H,s),7.69(1H,ddd,*J*=8.0,1.3,0.5Hz),^13^CNMR(400MHz,DMSOd6):178.48,162.83,158.97,157.28,156.36,145.30,135.72,132.35,130.06,127.11,121.80,121.39,113.58,109.80,106.79,105.79,93.52,56.41,56.23,55.74,52.53,50.82,49.81,28.49,11.73, MS (APCI): *m/z* 422.51 [M+1]^+^ (100%).

(2Z)-2-(2-bromobenzylidene)-7-(1-ethyl-1,2,3,6-tetrahydropyridin-4-yl)-4,6-dimethoxybenzofuran-3(2H)-one **(NEA3)** C_24_H_24_BrNO_4_,pale white powder, yield 55%, UV: λmax (MeOH) 395.22 nm (ε3.9900x10^3^cm^-1^M^-1^). ^1^H NMR: δ 0.98 (3H, t, *J* = 4.8 Hz), 2.34 (2H, ddd, *J* = 14.9, 10.1, 3.8 Hz), 2.65 (2H, q, *J* = 4.8 Hz), 2.74 (2H, ddd, *J* = 11.6, 3.8, 2.0 Hz), 3.13 (2H, dd, *J* = 13.9, 3.6 Hz), 3.82-3.83 (6H, 3.82 (s), 3.82 (s)), 6.03 (1H, dd, *J* = 5.1, 3.6 Hz), 6.23 (1H, s), 7.22 (1H, ddd, *J* = 8.1, 7.3, 1.7 Hz), 7.41-7.59 (4H, 7.45 (ddd, *J* = 8.2, 1.7, 0.5 Hz), 7.48 (s),7.50(ddd,*J*=8.2,7.3,1.5Hz),7.56(ddd,*J*=8.1,1.5,0.5Hz),^13^CNMR(400MHz,DMSOd6):178.46,162.83,158.97,157.28,144.02,135.72,134.16,132.23,132.15,129.88,126.64,126.11,121.39,109.80,107.18,105.79,93.52,56.42,56.22,52.53,50.82,49.81,28.49,11.73, MS (APCI): *m/z* 471.42 [M+1]^+^ (100%).

(2Z)-2-(4-bromobenzylidene)-7-(1-ethyl-1,2,3,6-tetrahydropyridin-4-yl)-4,6-dimethoxybenzofuran-3(2H)-one **(NEA4)** C_24_H_24_BrNO_4_**,**Orange crystals, yield 75%, UV: λmax (MeOH) 392.33 nm (ε3.5554x10^4^cm^-1^M^-1^ ). ^1^H NMR: δ 0.98 (3H, t, *J* = 4.8 Hz), 2.34 (2H, ddd, *J* = 14.9, 10.1, 3.8 Hz), 2.65 (2H, q, *J* = 4.8 Hz), 2.74 (2H, ddd, *J* = 11.6, 3.8, 2.0 Hz), 3.13 (2H, dd, *J* = 13.9, 3.6 Hz), 3.82-3.83 (6H, 3.82 (s), 3.82 (s)), 6.03 (1H, dd, *J* = 5.1, 3.6 Hz), 6.23 (1H, s), 7.35 (1H,s),7.45(2H,ddd, *J* =8.7,1.6,0.5Hz),7.62(2H,ddd,*J*=8.7,1.7,0.5Hz),^13^CNMR(400MHz,DMSOd6):179.03,162.81,158.97,157.28,143.40,135.72,132.20,131.76,123.44,121.39,112.03,109.80,105.77,93.52,56.41,56.23,52.53,50.82,49.81,28.49,11.73, MS (APCI): *m/z* 471.42 [M+1]^+^ (100%).

(2Z)-2-(3,4-dimethoxybenzylidene)-7-(1-ethyl-1,2,3,6-tetrahydropyridin-4-yl)-4,6-dimethoxybenzofuran-3(2H)-one **(NEA5)** C_26_H_29_NO_6_**,**yellow crystals, yield 69%,UV: λmax (MeOH) 392 nm ( ε3.9678x10^3^cm^-1^M^-1^). ^1^H NMR: δ 0.98 (3H, t, *J* = 4.8 Hz), 2.34 (2H, ddd, *J* = 14.9, 10.1, 3.8 Hz), 2.65 (2H, q, *J* = 4.8 Hz), 2.74 (2H, ddd, *J* = 11.6, 3.8, 2.0 Hz), 3.13 (2H, dd, *J* = 13.9, 3.7 Hz), 3.81-3.83 (9H, 3.82 (s), 3.81 (s), 3.82 (s)), 3.86 (3H, s), 6.03 (1H, dd, *J* = 5.1, 3.7 Hz), 6.23 (1H, s), 6.71 (1H, dd, *J* = 8.4, 0.4 Hz), 7.29 (1H, s), 7.33 (1H, dd, *J* = 1.9, 0.4 Hz), 7.65 (1H,dd,*J*=8.4,1.9Hz),^13^CNMR(400MHz,DMSOd6):179.04,162.81,158.97,157.28,150.24,149.65,143.68,135.72,127.31,127.18,121.39,114.10,112.11,111.45,109.81,105.77,93.52,56.41,56.23,55.90,55.88,52.53,50.82,49.81,28.49,11.73, MS (APCI): *m/z* 452.50 [M+1]^+^ (100%).

(2Z)-2-(4-chlorobenzylidene)-7-(1-ethyl-1,2,3,6-tetrahydropyridin-4-yl)-4,6-dimethoxybenzofuran-3(2H)-one **(NEA6)** C_24_H_24_ClNO_4_,yellow crystals, yield 73% ,UV: λmax (MeOH) 384nm ( ε3.5550x10^3^cm^-1^M^-1^ ). ^1^H NMR: δ 0.98 (3H, t, *J* = 4.8 Hz), 2.34 (2H, ddd, *J* = 14.9, 10.1, 3.8 Hz), 2.65 (2H, q, *J* = 4.8 Hz), 2.74 (2H, ddd, *J* = 11.6, 3.8, 2.0 Hz), 3.13 (2H, dd, *J* = 13.9, 3.6 Hz), 3.82-3.83 (6H, 3.82 (s), 3.82 (s)), 6.03 (1H, dd, *J* = 5.1, 3.6 Hz), 6.23 (1H, s), 7.35 (1H,s),7.51(2H,ddd, *J* =8.2,1.3,0.5Hz),7.63(2H,ddd,*J*=8.2,1.6,0.5Hz),^13^CNMR(400MHz,DMSOd6):179.03,162.81,158.97,157.28,143.40,135.72,133.75,131.97,131.06,129.06,121.39,111.91,109.80,105.77,93.52,56.41,56.23,52.53,50.82,49.81,28.49,11.73, MS (APCI): *m/z* 427.12 [M+1]^+^ (100%).

(2Z)-7-(1,2,3,6-tetrahydro-1-propylpyridin-4-yl)-4,6-dimethoxy-2-((naphthalen-3-yl)methylene)benzofuran-3(2H)-one **(NPA1)** C_29_H_29_NO_4_, light yellow powder, yield 58%, UV: λmax (MeOH) 386.76 nm (ε4.7543x10^3^cm^-1^M^-1^). ^1^H NMR: δ 0.92 (3H, t, *J* = 7.6 Hz), 1.58 (2H, qt, *J* = 7.6, 2.7 Hz), 2.34 (2H, ddd, *J* = 14.9, 10.1, 3.8 Hz), 2.50 (2H, t, *J* = 2.7 Hz), 2.74 (2H, ddd, *J* = 13.8, 3.8, 2.0 Hz), 3.08 (2H, dd, *J* = 13.9, 4.0 Hz), 3.81-3.83 (6H, 3.82 (s), 3.82 (s)), 6.04 (1H, dd, *J* = 5.6, 4.0 Hz), 6.23 (1H, s), 7.50 (1H, dddd, *J* = 8.0, 6.9, 1.9, 0.4 Hz), 7.59 (1H, dddt, *J* = 8.4, 2.5, 0.5, 0.4 Hz), 7.60-7.70 (2H, 7.64 (dddd, *J* = 8.0, 6.9, 2.6, 0.4 Hz), 7.67 (ddd, *J* = 8.4, 2.0, 0.4 Hz)), 7.70-7.84 (4H, 7.75 (s), 7.73 (dddt, *J* = 8.0, 2.1, 1.9, 0.5 Hz), 7.78 (dtq, *J* = 8.0, 2.5, 0.4 Hz),7.83(ddq, *J* =2.1,2.0,0.4Hz),^13^CNMR(400MHz,DMSOd6):178.53,162.83,158.97,157.28,143.17,135.72,134.29,133.85,131.24,130.45,127.94,127.71,126.52,126.49,125.70,121.36,109.80,105.79,105.73,93.52,58.58,56.41,56.23,53.16,51.67,28.47,20.36,11.91, MS (APCI): *m/z* 456.52 [M+1]^+^ (100%).

(2Z)-2-(4-(dimethylamino)benzylidene)-7-(1,2,3,6-tetrahydro-1-propylpyridin-4-yl)-4,6-dimethoxybenzofuran-3(2H)-one **(NPA2)** C_27_H_32_N_2_O_4_, pale yellow crystals, yield 60%, UV: λmax (MeOH) 411 nm (ε3.8543x10^3^cm^-1^M^-1^ ). ^1^H NMR: δ 0.92 (3H, t, *J* = 7.6 Hz), 1.58 (2H, qt, *J* = 7.6, 2.7 Hz), 2.34 (2H, ddd, *J* = 14.9, 10.1, 3.8 Hz), 2.50 (2H, t, *J* = 2.7 Hz), 2.74 (2H, ddd, *J* = 13.8, 3.8, 2.0 Hz), 2.87 (6H, s), 3.08 (2H, dd, *J* = 13.9, 3.8 Hz), 3.81-3.82 (6H, 3.82 (s), 3.82 (s)), 6.04 (1H, dd, *J* = 5.4, 3.8 Hz), 6.22 (1H, s), 6.72 (2H, ddd, *J* = 8.1, 1.1, 0.5 Hz), 7.27 (1H, s), 7.49 (2H,ddd,*J*=8.1,1.5,0.5Hz),^13^CNMR(400MHz,DMSOd6):179.03,162.81,158.97,157.28,151.70,143.40,135.72,131.64,124.58,121.36,112.21,112.03,109.80,105.77,93.52,58.58,56.41,56.23,53.16,51.67,40.27,28.47,20.36,11.91, MS (APCI): *m/z* 449.62 [M+1]^+^ (100%).

(2Z)-2-(2-bromobenzylidene)-7-(1,2,3,6-tetrahydro-1-propylpyridin-4-yl)-4,6-dimethoxybenzofuran-3(2H)-one **(NPA3)** C_25_H_26_BrNO_4_**,**light brown crystals, yield 54%, UV: λmax (MeOH) 407.65nm (ε3.6643x10^3^cm^-1^M^-1^). ^1^H NMR: δ 0.92 (3H, t, *J* = 7.6 Hz), 1.58 (2H, qt, *J* = 7.6, 2.7 Hz), 2.34 (2H, ddd, *J* = 14.9, 10.1, 3.8 Hz), 2.50 (2H, t, *J* = 2.7 Hz), 2.74 (2H, ddd, *J* = 13.8, 3.8, 2.0 Hz), 3.08 (2H, dd, *J* = 13.9, 3.9 Hz), 3.82-3.83 (6H, 3.82 (s), 3.82 (s)), 6.04 (1H, dd, *J* = 5.5, 3.9 Hz), 6.23 (1H, s), 7.22 (1H, ddd, *J* = 8.1, 7.3, 1.7 Hz), 7.41-7.59 (4H, 7.45 (ddd, *J* = 8.2, 1.7, 0.5 Hz), 7.48 (s), 7.50 (ddd, *J* = 8.2, 7.3, 1.5 Hz), 7.56 (ddd, *J* = 8.1, 1.5, 0.5 Hz),^13^CNMR(400MHz,DMSOd6):178.46,162.83,158.97,157.28,144.02,135.72,134.16,132.23,132.15,129.88,126.64,126.11,121.36,109.80,107.18,105.79,93.52,58.58,56.41,56.23,53.16,51.67,28.47,20.36,11.91, MS (APCI): *m/z* 486.52 [M+2]^+^ (100%).

(2Z)-2-(4-chlorobenzylidene)-7-(1,2,3,6-tetrahydro-1-propylpyridin-4-yl)-4,6-dimethoxybenzofuran-3(2H)-one **(NPA4)** C_25_H_26_ClNO_4_**,**light yellow crystals**,** yield 73%**,** UV: λmax (MeOH) 405 nm (ε4.8665x10^3^cm^-1^M^-1^ ). ^1^H NMR: δ 0.92 (3H, t, *J* = 7.6 Hz), 1.58 (2H, qt, *J* = 7.6, 2.7 Hz), 2.34 (2H, ddd, *J* = 15.0, 10.1, 3.8 Hz), 2.50 (2H, t, *J* = 2.7 Hz), 2.74 (2H, ddd, *J* = 13.8, 3.8, 2.0 Hz), 3.08 (2H, dd, *J* = 13.9, 3.9 Hz), 3.82-3.83 (6H, 3.82 (s), 3.82 (s)), 6.04 (1H, dd, *J* = 5.5, 3.9 Hz), 6.23 (1H, s), 7.35 (1H, s), 7.51 (2H, ddd,*J*=8.2,1.3,0.5Hz),7.63(2H,ddd,*J*=8.2,1.6,0.5Hz),^13^CNMR(400MHz,DMSOd6):179.04,162.81,158.97,157.28,143.96,135.72,133.63,133.27,132.53,132.06,130.10,130.08,121.36,111.54,109.80,105.77,93.52,58.58,56.41,56.23,53.16,51.67,28.47,20.36,11.91, MS (APCI): *m/z* 440.34 [M+1]^+^ (100%).

(2Z)-2-(3,4-dimethoxybenzylidene)-7-(1,2,3,6-tetrahydro-1-propylpyridin-4-yl)-4,6-dimethoxybenzofuran-3(2H)-one **(NPA5)** C_27_H_31_NO_6_, yellow crystals, yield 70%, UV: λmax (MeOH) 432.44nm (ε3.6754x10^3^cm^-1^M^-1^). ^1^H NMR: δ 0.92 (3H, t, *J* = 7.6 Hz), 1.58 (2H, qt, *J* = 7.6, 2.7 Hz), 2.34 (2H, ddd, *J* = 14.9, 10.1, 3.8 Hz), 2.50 (2H, t, *J* = 2.7 Hz), 2.74 (2H, ddd, *J* = 13.8, 3.8, 2.0 Hz), 3.08 (2H, dd, *J* = 13.9, 3.9 Hz), 3.81-3.83 (9H, 3.82 (s), 3.81 (s), 3.82 (s)), 3.86 (3H, s), 6.04 (1H, dd, *J* = 5.5, 3.9 Hz), 6.23 (1H, s), 6.71 (1H, dd, *J* = 8.4, 0.4 Hz), 7.29 (1H, s), 7.33(1H,dd,*J*=1.9,0.4Hz),7.65(1H,dd, *J* =8.4,1.9Hz),^13^CNMR(400MHz,DMSOd6):179.04,162.81,158.97,157.28,150.24,149.65,143.68,135.72,127.31,127.18,121.36,114.10,112.11,111.45,109.81,105.77,93.52,58.58,56.41,56.23,55.90,55.88,53.16,51.67,28.47,20.36,11.9,MS(APCI):*m/z*466.61 [M+1]^+^ (100%).

2Z)-7-(1,2,3,6-tetrahydro-1-isopropylpyridin-4-yl)-4,6-dimethoxy-2-((naphthalen-3 yl)methylene)benzofuran-3(2H)-one **(NISOA1)** C_29_H_29_NO_4_**,** pale white crystals**,** yield 77%, UV: λmax (MeOH) 378.61nm (ε3.9876x10^3^cm^-1^M^-1^). ^1^H NMR: δ 1.15 (6H, d, *J* = 6.9 Hz), 2.33 (2H, ddd, *J* = 14.9, 10.1, 3.8 Hz), 2.71 (2H, ddd, *J* = 11.7, 3.8, 2.0 Hz), 3.05-3.18 (3H, 3.13 (dd, *J* = 13.9, 3.9 Hz), 3.07 (sept, *J* = 6.9 Hz)), 3.81-3.83 (6H, 3.82 (s), 3.82 (s)), 6.04 (1H, dd, *J* = 5.8, 3.9 Hz), 6.23 (1H, s), 7.50 (1H, dddd, *J* = 8.0, 6.9, 1.9, 0.4 Hz), 7.59 (1H, dddt, *J* = 8.4, 2.5, 0.5, 0.4 Hz), 7.60-7.70 (2H, 7.64 (dddd, *J* = 8.0, 6.9, 2.6, 0.4 Hz), 7.67 (ddd, *J* = 8.4, 2.0, 0.4 Hz)), 7.70-7.84 (4H, 7.75 (s), 7.73 (dddt, *J* = 8.0, 2.1, 1.9, 0.5 Hz), 7.78 (dtq, *J* = 8.0, 2.5, 0.4 Hz), 7.83 (ddq,*J*=2.1,2.0,0.4Hz),^13^CNMR(400MHz,DMSOd6):178.53,162.83,158.97,157.28,143.17,135.72,134.29,133.85,131.24,130.45,127.94,127.71,126.52,126.49,125.70,121.13,109.81,105.79,105.73,93.52,56.41,56.23,53.62,52.36,49.36,28.55,19.29, MS (APCI): *m/z* 456.61 [M+1]^+^ (100%).

(2Z)-2-(2-bromobenzylidene)-7-(1,2,3,6-tetrahydro-1-isopropylpyridin-4-yl)-4,6-dimethoxybenzofuran-3(2H)-one **(NISOA2)** C_25_H_26_BrNO_4_**,** white powder**,** yield 67% UV: λmax (MeOH) 322.78 nm (ε4.0087x10^4^cm^-1^M^-1^). ^1^H NMR: δ 1.15 (6H, d, *J* = 6.9 Hz), 2.33 (2H, ddd, *J* = 15.0, 10.1, 3.8 Hz), 2.71 (2H, ddd, *J* = 11.7, 3.8, 2.0 Hz), 3.05-3.18 (3H, 3.13 (dd, *J* = 13.9, 3.8 Hz), 3.07 (sept, *J* = 6.9 Hz)), 3.82-3.83 (6H, 3.82 (s), 3.82 (s)), 6.04 (1H, dd, *J* = 5.7, 3.8 Hz), 6.23 (1H, s), 7.22 (1H, ddd, *J* = 8.1, 7.3, 1.7 Hz), 7.41-7.59 (4H, 7.45 (ddd, *J* = 8.2, 1.7, 0.5 Hz), 7.48 (s),7.50(ddd, *J* =8.2,7.3,1.5Hz),7.56(ddd,*J*=8.1,1.5,0.5Hz),^13^CNMR(400MHz,DMSOd6):178.46,162.83,158.97,157.28,144.02,135.72,134.16,132.23,132.15,129.88,126.64,126.11,121.13,109.80,107.18,105.79,93.52,56.41,56.23,53.62,52.36,49.36,28.55,19.29, MS (APCI): *m/z* 486.32 [M+2]^+^ (100%).

2Z)-2-(2-methylbenzylidene)-7-(1,2,3,6-tetrahydro-1-isopropylpyridin-4-yl)-4,6-dimethoxybenzofuran-3(2H)-one **(NISOA3)** C_26_H_29_NO_4_,slight yellow crystals, yield 77%, UV: λmax (MeOH) 381.22 nm (ε3.9987x10^3^cm^-1^M^-1^). ^1^H NMR: δ 1.15 (6H, d, *J* = 6.9 Hz), 2.25-2.41 (5H, 2.26 (s), 2.34 (ddd, *J* = 15.0, 10.1, 3.8 Hz)), 2.71 (2H, ddd, *J* = 11.7, 3.8, 2.0 Hz), 3.05-3.18 (3H, 3.13 (dd, *J* = 13.9, 3.8 Hz), 3.07 (sept, *J* = 6.9 Hz)), 3.82-3.83 (6H, 3.82 (s), 3.82 (s)), 6.04 (1H, dd, *J* = 5.7, 3.8 Hz), 6.23 (1H, s), 7.04-7.24 (3H, 7.09 (ddd, *J* = 7.9, 7.7, 2.6 Hz), 7.13 (ddd, *J* = 7.9, 7.7, 1.4 Hz), 7.21 (ddd, *J* = 7.9, 2.6, 0.4 Hz)), 7.46-7.52 (2H, 7.49 (ddd, *J* = 7.9, 1.4, 0.4 Hz), 7.49(s),^13^CNMR(400MHz,DMSOd6):178.40,162.83,158.97,157.28,142.47,138.86,135.72,133.45,131.76,130.06,129.80,125.53,121.13,109.80,107.09,105.79,93.52,56.41,56.23,53.62,52.36,49.36,28.55,19.29, MS (APCI): *m/z* 420.60 [M+1]^+^ (100%).

(2Z)-2-(3,4-dimethoxybenzylidene)-7-(1,2,3,6-tetrahydro-1-isopropylpyridin-4-yl)-4,6-dimethoxybenzofuran-3(2H)-one **(NISOA4)** C_27_H_31_NO_6_, yellow crystals, yield 64%, UV: λmax (MeOH) 429.09 nm (ε3.7790x10^3^cm^-1^M^-11^H NMR: δ 1.15 (6H, d, *J* = 6.9 Hz), 2.34 (2H, ddd, *J* = 15.0, 10.1, 3.8 Hz), 2.71 (2H, ddd, *J* = 11.7, 3.8, 2.0 Hz), 3.05-3.18 (3H, 3.13 (dd, *J* = 13.9, 3.8 Hz), 3.07 (sept, *J* = 6.9 Hz)), 3.81-3.83 (9H, 3.82 (s), 3.81 (s), 3.82 (s)), 3.86 (3H, s), 6.04 (1H, dd, *J* = 5.7, 3.8 Hz), 6.23 (1H, s), 6.71 (1H, dd, *J* = 8.4, 0.4 Hz), 7.29 (1H, s), 7.33 (1H, dd, *J* = 1.9,0.4Hz),7.65(1H,dd, *J* =8.4,1.9Hz),^13^CNMR(400MHz,DMSOd6):179.04,162.81,158.97,157.28,150.24,149.65,143.68,135.72,127.31,127.18,121.13,114.10,112.11,111.45,109.80,105.77,93.52,56.41,56.23,55.90,55.88,53.62,52.36,49.36,28.55,19.29, ). MS (APCI): *m/z* 466.62 [M+1]+ (100%).

(2Z)-2-(4-methoxybenzylidene)-7-(1,2,3,6-tetrahydro-1-isopropylpyridin-4-yl)-4,6-dimethoxybenzofuran-3(2H)-one **(NISOA5)** C_26_H_29_NO_5_, light yellow crystals, yield 65%, UV: λmax (MeOH) 416.98 nm (ε4.9876x10^3^cm^-1^M^-1^). ^1^H NMR: δ 1.15 (6H, d, *J* = 6.9 Hz), 2.34 (2H, ddd, *J* = 15.0, 10.1, 3.8 Hz), 2.71 (2H, ddd, *J* = 11.6, 3.8, 2.0 Hz), 3.05-3.18 (3H, 3.13 (dd, *J* = 13.9, 3.8 Hz), 3.07 (sept, *J* = 6.9 Hz)), 3.79 (3H, s), 3.81-3.82 (6H, 3.82 (s), 3.82 (s)), 6.04 (1H, dd, *J* = 5.7, 3.8 Hz), 6.23 (1H, s), 7.22 (2H, ddd, *J* = 8.8, 1.2, 0.4 Hz), 7.32 (1H, s), 7.79 (2H, ddd,*J*=8.8,1.7,0.4Hz),^13^CNMR(400MHz,DMSOd6):179.03,162.81,160.43,158.97,157.28,143.40,135.72,132.39,125.93,121.13,113.69,111.95,109.80,105.77,93.52,56.41,56.23,55.32,53.62,52.36,49.36,28.55,19.29, MS (APCI): *m/z* 436.52 [M+1]+ (100%).

(2Z)-2-(4-chlorobenzylidene)-7-(1,2,3,6-tetrahydro-1-isopropylpyridin-4-yl)-4,6-dimethoxybenzofuran-3(2H)-one **(NISOA6)** C_25_H_2_ClNO_4_,yellow crystals, yield 62%, UV: λmax (MeOH) 401.89 nm (ε4.8876x10^3^cm^-1^M^-1^). ^1^H NMR: δ 1.15 (6H, d, *J* = 6.9 Hz), 2.34 (2H, ddd, *J* = 15.0, 10.1, 3.8 Hz), 2.71 (2H, ddd, *J* = 11.6, 3.8, 2.0 Hz), 3.05-3.18 (3H, 3.13 (dd, *J* = 13.9, 3.8 Hz), 3.07 (sept, *J* = 6.9 Hz)), 3.82-3.83 (6H, 3.82 (s), 3.82 (s)), 6.04 (1H, dd, *J* = 5.7, 3.8 Hz), 6.23 (1H, s), 7.35 (1H, s), 7.51 (2H, ddd, *J* = 8.2, 1.3, 0.5 Hz), 7.63 (2H, ddd, *J* = 8.2, 1.6, 0.5 Hz), ^13^CNMR(400MHz,DMSOd6):179.03,162.81,158.97,157.28,143.40,135.72,133.75,131.97,131.06,129.06,121.13,111.91,109.80,105.77,93.52,56.41,56.23,53.62,52.36,49.36,28.55,19.29,MS (APCI): *m/z* 441.12 [M+1]^+^ (100%).

Structure of Synthesized test compounds

| **S.No.** | **Code** | **Structure** |
| --- | --- | --- |
| 1 | **NMA1** |  |
| 2 | **NMA2** |  |
| 3 | **NMA3** |  |
| 4 | **NMA4** |  |
| 5 | **NMA5** |  |
| 6 | **NMA6** |  |
| 7 | **NEA1** |  |
| 8 | **NEA2** |  |
| 9 | **NEA3** |  |
| 10 | **NEA4** |  |
| 11 | **NEA5** |  |
| 12 | **NEA6** |  |
| 13 | **NPA1** |  |
| 14 | **NPA2** |  |
| 15 | **NPA3** |  |
| 16 | **NPA4** |  |
| 17 | **NPA5** |  |
| 18 | **NISOA1** |  |
| 19 | **NISOA2** |  |
| 20 | **NISOA3** |  |
| 21 | **NISOA4** |  |
| 22 | **NISOA5** |  |
| 23 | **NISOA6** |  |
